# Supplementary material for: FBXL21 regulates diurnal proteostasis in skeletal muscle by targeting DNAJB6 and client proteins
Source: EMBO Rep. 2026 Sep 30;27(21):6366–95. doi: 10.1038/s44319-026-00922-1 (PMC13743554; doi:10.1038/s44319-026-00922-1)
Supplement: Supplementary file 1 [file 44319_2026_922_MOESM1_ESM.pdf]

EMBO reports

FBXL21 regulates diurnal proteostasis in skeletal muscle by targeting DNAJB6 and client proteins

Zheng Chen, Ji Ye Lim, Chorong Han, Sun Young Kim, Sehyun Jung, Kristin Eckel-Mahan, Sung Yun Jung, Karyn Esser, Seung-Hee Yoo, Marvin Wirianto, Jaebok Wi, and Jane Nguyen

Corresponding author(s): Seung-Hee Yoo (Seung-Hee.Yoo@uth.tmc.edu)

Review Timeline:

Submission Date:	23rd Oct 25
Editorial Decision:	4th Dec 25
Revision Received:	17th Mar 26
Editorial Decision:	9th Jul 26
Revision Received:	11th Aug 26
Accepted:	20th Aug 26

Editor: Martina Rembold

Transaction Report:

(Note: With the exception of the correction of typographical or spelling errors that could be a source of ambiguity, letters and reports are not edited. Depending on transfer agreements, referee reports obtained elsewhere may or may not be included in this compilation. Referee reports are anonymous unless the Referee chooses to sign their reports.)

Dear Dr. Yoo

Thank you for r the submission of your research manuscript to our journal. We have now received the full set of referee reports that is copied below.

As you will see, the referees acknowledge that the findings are potentially interesting, but they also raise a number of concerns and have suggestions how to further strengthen the data.

I think that all concerns from the referees are pertinent and should be addressed. I would like to comment here on two points raised by the referees: Please clearly indicate whether n = 3 refers to technical or independent replicates. If these are technical only, no statistics can be applied, as the number of experiments is in fact one. Please also consider comment 1 from referee #2 about the integration of other E3 ligases in Desmin stability. I think it is important to put your findings into context and maybe even test the contribution of MuRF1 and Trim32 in your context.

I would also recommend citing Hirano et al, Cell 2013 (23452856), which appears to have also reported on FBXL21's role in circadian proteostasis, similar to Yoo et al, Cell 2013, if I am not mistaken.

Given these constructive comments, we would like to invite you to revise your manuscript with the understanding that the referee concerns (as detailed above and in their reports) must be fully addressed and their suggestions taken on board. Please address all referee concerns in a complete point-by-point response. Acceptance of the manuscript will depend on a positive outcome of a second round of review. It is EMBO Reports policy to allow a single round of revision only and acceptance or rejection of the manuscript will therefore depend on the completeness of your responses included in the next, final version of the manuscript.

We realize that it is difficult to revise to a specific deadline. In the interest of protecting the conceptual advance provided by the work, we recommend a revision within 3 months (March 4th, 2026). Please discuss the revision progress ahead of this time with the editor if you require more time to complete the revisions.

I am also happy to discuss the revision further via e-mail or a video call, if you wish.

You can either publish the study as a short report or as a full article. For short reports, the revised manuscript should not exceed 27,000 characters (including spaces but excluding materials & methods and references) and 5 main plus 5 expanded view figures. The results and discussion sections must further be combined, which will help to shorten the manuscript text by eliminating some redundancy that is inevitable when discussing the same experiments twice. For a normal article there are no length limitations, but it should have more than 5 main figures and the results and discussion sections must be separate. In both cases, the entire materials and methods must be included in the main manuscript file.

================
IMPORTANT NOTE:

We perform an initial quality control of all revised manuscripts before re-review. Your manuscript will FAIL this control and the handling will be delayed IN CASE the following APPLIES:

1) A data availability section providing access to data deposited in public databases is missing. If you have not deposited any data, please add a sentence to the data availability section that explains that.

2) Your manuscript contains statistics and error bars based on n=2. Please use scatter blots in these cases. No statistics should be calculated if n=2.

When submitting your revised manuscript, please carefully review the instructions that follow below. Failure to include requested items will delay the evaluation of your revision.

================

When submitting your revised manuscript, we will require:

1) a .docx formatted version of the manuscript text (including legends for main figures, EV figures and tables). Please make sure that the changes are highlighted to be clearly visible.

2) individual production quality figure files as .eps, .tif, .jpg (one file per figure).
Please download our Figure Preparation Guidelines (figure preparation pdf) from our Author Guidelines pages

https://www.embopress.org/page/journal/14693178/authorguide for more info on how to prepare your figures.

3) a .docx formatted letter INCLUDING the reviewers' reports and your detailed point-by-point responses to their comments. As part of the EMBO Press transparent editorial process, the point-by-point response is part of the Review Process File (RPF), which will be published alongside your paper.

4) a complete author checklist, which you can download from our author guidelines (<https://www.embopress.org/page/journal/14693178/authorguide>). Please insert information in the checklist that is also reflected in the manuscript. The completed author checklist will also be part of the RPF.

5) Please note that all corresponding authors are required to supply an ORCID ID for their name upon submission of a revised manuscript (<https://orcid.org/>). Please find instructions on how to link your ORCID ID to your account in our manuscript tracking system in our Author guidelines (<https://www.embopress.org/page/journal/14693178/authorguide#authorshipguidelines>)

6) We replaced Supplementary Information with Expanded View (EV) Figures and Tables that are collapsible/expandable online. A maximum of 5 EV Figures can be typeset. EV Figures should be cited as 'Figure EV1, Figure EV2" etc... in the text and their respective legends should be included in the main text after the legends of regular figures.

- For the figures that you do NOT wish to display as Expanded View figures, they should be bundled together with their legends in a single PDF file called *Appendix*, which should start with a short Table of Content. Appendix figures should be referred to in the main text as: "Appendix Figure S1, Appendix Figure S2" etc. See detailed instructions regarding expanded view here: <https://www.embopress.org/page/journal/14693178/authorguide#expandedview>

- Additional Tables/Datasets should be labeled and referred to as Table EV1, Dataset EV1, etc. Legends have to be provided in a separate tab in case of .xls files. Alternatively, the legend can be supplied as a separate text file (README) and zipped together with the Table/Dataset file.

7) Before submitting your revision, primary datasets (and computer code, where appropriate) produced in this study need to be deposited in an appropriate public database (see < https://www.embopress.org/page/journal/14693178/authorguide#dataavailability>).
Specifically, we would kindly ask you to provide public access to the mass spectrometry dataset.

Please remember to provide a reviewer password if the datasets are not yet public.

The accession numbers and database should be listed in a formal "Data Availability " section (placed after Materials & Method) that follows the model below (see also < https://www.embopress.org/page/journal/14693178/authorguide#dataavailability>). Please note that the Data Availability Section is restricted to new primary data that are part of this study.

Data availability

The datasets (and computer code) produced in this study are available in the following databases:

- RNA-Seq data: Gene Expression Omnibus GSE46843 (https://www.ncbi.nlm.nih.gov/geo/query/acc.cgi?acc=GSE46843)
- [data type]: [name of the resource] [accession number/identifier/doi] ([URL or identifiers.org/DATABASE:ACCESSION])

*** Note - All links should resolve to a page where the data can be accessed. ***

8) At EMBO Press we ask authors to provide source data for the main manuscript figures. You will receive a separate email with instructions for providing source data with your revised manuscript, including how to upload and organize the files.

Additional information on source data and instruction on how to label the files are available <https://www.embopress.org/page/journal/14693178/authorguide#sourcedata>

9) The journal requires a statement specifying whether or not authors have competing interests (defined as all potential or actual interests that could be perceived to influence the presentation or interpretation of an article). In case of competing interests, this must be specified in your disclosure statement. Further information: https://www.embopress.org/competing-interests

10) Figure legends and data quantification:
The following points must be specified in each figure legend:

- the name of the statistical test used to generate error bars and P values,
- the EXACT p-values,

- the number (n) of independent experiments (please specify technical or biological replicates) underlying each data point,
- the nature of the bars and error bars (s.d., s.e.m.)

- If the data are obtained from n {less than or equal to} 5, show the individual data points in addition to the SD or SEM.
- If the data are obtained from n {less than or equal to} 2, use scatter blots showing the individual data points.

Discussion of statistical methodology can be reported in the materials and methods section, but figure legends should contain a basic description of n, P and the test applied.

See also the guidelines for figure legend preparation:
https://www.embopress.org/page/journal/14693178/authorguide#figureformat

- Please also include scale bars in all microscopy images and define their size in the legend, not the image.

11) Our journal encourages inclusion of *data citations in the reference list* to directly cite datasets that were re-used and obtained from public databases. Data citations in the article text are distinct from normal bibliographical citations and should directly link to the database records from which the data can be accessed. In the main text, data citations are formatted as follows: "Data ref: Smith et al, 2001" or "Data ref: NCBI Sequence Read Archive PRJNA342805, 2017". In the Reference list, data citations must be labeled with "[DATASET]". A data reference must provide the database name, accession number/identifiers and a resolvable link to the landing page from which the data can be accessed at the end of the reference. Further instructions are available at <https://www.embopress.org/page/journal/14693178/authorguide#referencesformat>.

12) All Materials and Methods need to be described in the main text using our 'Structured Methods' format. According to this format, the Methods section includes a Reagents and Tools Table (listing key reagents, experimental models, software and relevant equipment and including their sources and relevant identifiers) followed by a Methods and Protocols section describing the methods, ideally using a step-by-step protocol format. The aim is to facilitate adoption of the methodologies across labs. Please download and fill our Reagents and Tools Table template (.docx), which you can find in our author guidelines: https://www.embopress.org/page/journal/14693178/authorguide#structuredmethods.
When submitting your revised manuscript, please do not include the Reagents and Tools Table in the Methods section of the manuscript but upload it as a separate file choosing the file type "Reagent Table".
An example of a Method paper with Structured Methods can be found here:
https://www.embopress.org/doi/10.15252/msb.20178071.

13) As part of the EMBO publication's Transparent Editorial Process, EMBO Reports publishes online a Review Process File to accompany accepted manuscripts. This File will be published in conjunction with your paper and will include the referee reports, your point-by-point response and all pertinent correspondence relating to the manuscript.

You are able to opt out of this by letting the editorial office know (emboreports@embo.org). If you do opt out, the Review Process File link will point to the following statement: "No Review Process File is available with this article, as the authors have chosen not to make the review process public in this case."

We would also welcome the submission of cover suggestions, or motifs to be used by our Graphics Illustrator in designing a cover.

I look forward to seeing a revised form of your manuscript when it is ready and please let me know if you have questions or comments regarding the revision.

Yours sincerely,

Martina Rembold, PhD
Senior Editor
EMBO reports

==================

Referee #1:

Review of "Fblx21 regulates circadian proteostasis and stress response by targeting DNAJB6 and client proteins" - Lim et al
The authors of this manuscript explore the potential mechanistic interaction of Fbxl21 and proteostasis in skeletal muscle. They

arrived at this project by finding a proteostasis component as an interactor of Fbxl21 in protein interaction screens, and focus the majority of experiments on identifying the potential interactions and roles of Fbxl21 this process. Overall the data presented are generally quite convincing (despite some potential caveats details below) and demonstrate that Fbxl21, as a ubiquitin ligase, regulates DnaJB6 stability along with a number of other proteostasis related components. The authors do a nice job of mixing both in vitro (hek293 cells), semi-in vivo (C2C12 cells) and some in vivo (mouse mutant) based experiments, that are all complementary. I did find a few areas that could be bolstered to enhance both presentation and overall sense of 'experimental rigor' that are noted below that are hopefully easy to address.
Minor points -
1) All the cell- culture/over expression assays should be referred to in the text as events that "can" happen... these experiments themselves do not tell us that these events "do" happen.
2) Panel 3D - the bottom 4 blots are "input"? Please label accordingly.
3) Starting in Fig 2, in co-transfection studies, the authors use the notation "construct 1/construct 2" - the "/" sign is confusing at first glance... changing it to a "+" could be clearer.
4) For the quantification of CHX experiments (e.g. fig 2b,c), it would be easier of reader if they included an indication of statistical sig between relevant groups on the figure itself, rather than burying this in the text of the legend.
5) Also, for these same experiments, please define 'n=3' - is this 3 replicates from a single experimental trial or 3 independent trials with a single replicate each? The methods description makes it sound like the former...if so, please add how many times each were replicated with similar results.
6) Fig 5a (and subsequent similar figs)- it is very difficult to see the labels within the images, with the label the same color as the staining.
7) Fig 5 and subsequent figs - please provide more details on the methods used in the quantification of IF images, with particular regard to ensuring readers are clear that these results are well controlled for any technical variances (IF procedures, imaging settings etc) that could directly and artificially impact these data.
8) The title indicates that Fbxl21 regulates "circadian proteostasis", however, the experiments presented do not demonstrate this - first, 2 timepoints is not enough "circadian" in my view, and second, most data do not indicate that the day-night different is eliminated, rather the PSTM mutant seem to be at an overall elevated levels (Figs 7-8) - thus not a "circadian" deficit by definition if the rhythm is still present.
9) I'm curious - to Fbxl21 mutants have physiological muscle deficits that are or could be attributed to altered proteostasis? I was surprised this was not addressed, at least speculatively, in the MS.

Referee #2:

The manuscript by Lim and colleagues sought to expand our knowledge of circadian proteostasis via discovery of the Fbxl21 interactome and how this E3 ubiquitin ligase might degrade and regulate proteins that are important for the stress response system. The authors utilized an array of in vitro and in vivo approaches to target the Fbxl21 interactome. Firstly, Yeast hybrid screens and affinity pull down followed by mass spectrometry analysis was used to identify Fbxl21 targets. The studies were followed up by using Fbxl21 KO c2c12 cells to assess the Fbxl21, DNAJB6 and HSP70 interactions. The authors also used a Fbxl21 hypomorph mutant model (psttm) to study interactors in the context of stress response via an exercise stimulus. The authors also exposed C2C12 cells to heat stress to investigate stress granule resolution and the formation of protein aggregates under Fbxl21 KO conditions. A major finding from the current study was the regulation of DNAJB6 via Fbxl21 and how this altered turnover of client proteins, desmin and TDP-43. Detailed below are my comments.

1) The authors identified the changes in desmin aggregation as an outcome measure for a client protein DNAJB6 (Fig 5). It is worth noting in the discussion that E3 ligases MuRF1 and Trim32 in skeletal muscle have also been highlighted to regulate Desmin and change its solubility (PMID38283190; PMID22908310; PMID34179788). This is important given that E3 ligases are suggested have only specific substrates and little appreciation is given to the fact that E3s may work in tandem to degrade mutual substrates (PMID37955121).
2) Did the Psttm mice display any exercise performance differences or the time to exhaustion between wild type and Psttm mice?
3) Fig 1F, how were aggregates for DNAJB6 defined in skeletal muscle fibers? Given the size and composition of the lateral and medial gastrocnemius muscle, was the aggregation spread across these muscles or localized to a particular area? Did the authors account for regionality in the quantification? The use of > 100 fibers as a threshold appears to be a low number given the makeup of the lateral and medial gastrocnemius muscle and its fiber density.

The same aspect in this comment can be applied for Fig 5A about the quantification of desmin inclusion bodies across the tissue.

4) Given Fbxl21 function is reliant on SCF Complex interaction, is this complex altered in the Psttm mice? Is DNAJB6 targeted degradation dependent not only on Fbxl21 but also the SCF complex itself?
5) It would be interesting to know if the changes in biochemical properties for stress granule formulation, TDP-43 and desmin aggregation leads to impaired skeletal muscle function in the current Psttm mice used? The authors highlight how these markers of proteotoxic stress contribute to other myopathies, yet discussion on skeletal muscle function in Psttm mice might benefit the

reader too. Protein quality control can be compromised in skeletal muscle via E3 ligase knockdown, yet muscle mass can remain intact (PMID33658508).

Referee #3:

The manuscript by Lim et al identifies an new interaction between FBXL21 and DNAJB6. Specifically, DNAJB6 is ubiquitinated by FBXL2 leading to its degradation however, when DNAJB6 recognizes client protein, this ubiquitination can then be transferred to the clients (Desmin and stress granule proteins) in a chaperone dependent manner akin to what is already established for proteins such as CHIP. Moreover, this occurs in circadian manner. The manuscript is conceptually interesting and proposes a novel mechanism for DNAJB6 mediated chaperone function but several issues remain.

1) The data in figure 1 demonstrates that DNAJB6 can associate with FBXL21 as found by unbiased IPs and yeast two hybrids. The increases in DNAJB6 in the nightime and with loss of FBXL21 could be due to a change in degradation but could also be due to increased gene expression. QPCR or other means of confirming that DNAJB6 isn't upregulated is necessary.

2) I am a bit confused by the claim in Figure 1D that their is reduced diurnal oscilalation of DNAJB6 in the Psttm KO mice. the DNAJB6b quantified change look greater in the Psttm mice to me.

3) The immunofluorscense in skeletal muscle in figures 1F, 7 and 8 are difficult to interpret. Moreover, there is not methodology for how quantitation was performed, how mouse muscle was fixed and sectioned. Relative fluorscence intensity does not seem appropriate (is it for an individual fiber? In the case of 1F,how is a DNAJB6 aggregate defined. Is DNAJB6 now insoluble via fractionation, ubiquitinated. The diurnal change is not very convincing via IF.

4) Figure 2 D and E would benefit from some molecular weight markers.

5) Figure 3 is an important figure. Does Fbxl21 associate with HSP70 independently of DNAJB6 or as a complex. Figure 1A would suggest it can occur indendently. Does Figure 3B and 3c have the levels of HSP70 and FBXl21 in the reciprocal treatments. Figure 3e should show that FBxl21 is similarly expressed in the control and chip SIRNA

6) In Figure 4, are LGMDD1 mutants not ubiquitinated? In addition, Figure 4C needs to show the input of DNAJB6 levels and expression levels of HSP70 in the tissue.

7) Figure 5A has similar issues regarding IF in skeletal muscle, these look oversaturated and how do we define and aggreagte. The IF images look like every fiber has an aggregate but the quantiation just says 2-6% of fibers. Does the desmin or DNAJB6 become insoluble. Figure 5B, it is difficult to know what is being quantitated. puncta/fluorescence intensity. Figure 5E is important and needs to show blots for all the transfected proteins to confirm they are equal across condidtions.

8) Figure 6 is not really showing dynamics, just formation of stress granules. and formation of TDP-43 cytosolic puncta. Not entirely clear what they are quantifiyning. Figure S5A no gapdh Figure S5C polyQ aggregates form on their own and not with heat shock. I am not certain what phenomenon is being measured. The control that is needed is a non expanded polyQ.

9) Figure 7 and 8 Suffer from similar issues. The reliance of IF and unclear quantitation based on relative fluorescent intensity. the paper calls these stress granules but not sure what they are quantitating. I am not certain how one can be certain these are stress granules as a stress granule is reversible and we don't know if these are aggregates or stress granules. Immunoblotting showing insolubilty or other markers would be helpful. Quantitation of loss of TDP-43 from the nuclear may be relevant or demonstration of cryptic splicing.

Overall, the initial biochemical studies show promise but the movement into the mouse muscle is challenged by unclear relevance of mean fluorescent intensity and lack of western confirmation. finally, the connection to circadian rhythem is weak and doesnt really add much.

Point-by-point response to peer review comments

We thank the Editor for thoughtful handling and the reviewers for expert comments regarding our manuscript (EMBOR-2025-63032V1). We greatly appreciate the overall positive assessments and numerous insightful suggestions. As detailed below, we have performed extensive experiments, leading to 8 new figure panels and 7 updated figure panels, and conducted additional analyses to address all review comments. We have revised the manuscript text and added references accordingly, in compliance with journal guidelines. Below we provide a detailed point-by-point response to the comments from the reviewers.

Reviewer 1 (R1): The authors of this manuscript explore the potential mechanistic interaction of Fbxl21 and proteostasis in skeletal muscle. They arrived at this project by finding a proteostasis component as an interactor of Fbxl21 in protein interaction screens, and focus the majority of experiments on identifying the potential interactions and roles of Fbxl21 this process. Overall, the data presented are generally quite convincing (despite some potential caveats details below) and demonstrate that Fbxl21, as a ubiquitin ligase, regulates DnaJB6 stability along with a number of other proteostasis related components. The authors do a nice job of mixing both in vitro (hek293 cells), semi-in vivo (C2C12 cells) and some in vivo (mouse mutant) based experiments, that are all complementary. I did find a few areas that could be bolstered to enhance both presentation and overall sense of 'experimental rigor' that are noted below that are hopefully easy to address.

Minor points

1. All the cell- culture/over expression assays should be referred to in the text as events that "can" happen... these experiments themselves do not tell us that these events "do" happen.
Response: We thank the reviewer for the thoughtful comments. In response to this point, we have revised the wording in relevant Results sections for more accurate interpretation. Specifically, we made changes in the description for the over-expression panels in Figs. 2, 3, 4, and 5. For example, on page 6, "Treatment with MG132, a 26S proteasome inhibitor, blocked FBXL21-mediated degradation of mouse DNAJB6b, suggesting that FBXL21 may promote the ubiquitination and proteasomal turnover of DNAJB6b (Fig. 2C). FBXL21 expression also increased the rate of human DNAJB6 degradation (Fig. EV2A,B). Interestingly, the GSK-3β inhibitor CHIR-99021 decelerated FBXL21-mediated degradation, suggesting that GSK-3β may regulate FBXL21-dependent DNAJB6b degradation similar to previously reported FBXL21 target substrates[19, 20] (Fig. 2C). In ubiquitination assays, both mouse DNAJB6 isoforms (Fig. 2D, 2E) and human DNAJB6 (Fig. EV2C,D) were efficiently ubiquitinated in the presence of FBXL21. Together, these results show that FBXL21 can promote DNAJB6 ubiquitination and its targeting to proteasomal degradation".

2. Panel 3D - the bottom 4 blots are "input"? Please label accordingly.
Response: That is correct. We have added "Input" in Panel 3D. Thank you.

3. Starting in Fig 2, in co-transfection studies, the authors use the notation "construct 1/construct 2" - the "/" sign is confusing at first glance... changing it to a "+" could be clearer.
Response: As suggested, we have replaced the '/' signs with '+' to improve clarity.

4. For the quantification of CHX experiments (e.g. fig 2b,c), it would be easier of reader if they included an indication of statistical sig between relevant groups on the figure itself, rather than burying this in the text of the legend.
Response: As suggested, we have included statistical significance between the relevant groups in the figure by comparing K, the rate constant representing the speed of decay.

5. Also, for these same experiments, please define 'n=3' - is 3 replicates from a single experimental trial or 3 independent trials with a single replicate each? The methods description makes it sound like the former...if so, please add how many times each were replicated with similar results.
Response: We thank the reviewer for raising this important point. In all cell studies, n = 3 refers to three independent biological replicates for each experiment which has also been repeated to ensure reproducibility. Specifically, in each independent experiment, three or more biological replicates were included, corresponding to separate wells undergoing parallel transfections, sample preparation, and western blot analysis. Each experiment was repeated for a total of three times (in some cases more). Similar results were obtained from the independent experiments, and representative data are shown in the figures. We have revised the Methods section (page 18) and figure legends to clarify this issue.

6. Fig 5a (and subsequent similar figs) - it is very difficult to see the labels within the images, with the label the same color as the staining.
Response: As suggested, the labels in Figures 5 and 6 have been repositioned, and the protein (antibody) names have been placed outside the images to improve visibility. Thank you.

7. Fig 5 and subsequent figs - please provide more details on the methods used in the quantification of IF images, with particular regard to ensuring readers are clear that these results are well controlled for any technical variances (IF procedures, imaging settings etc) that could directly and artificially impact these data.
Response: We have now added detailed descriptions in the Methods section regarding the quantification of immunofluorescence (IF) images, including how we quantified Desmin aggregates and how technical variables such as imaging settings were controlled to ensure reliable results.
Specifically, DNAJB6 and Desmin aggregates are defined as well-demarcated, clear puncta or inclusion-like cytoplasmic structures distinguishable from the surrounding diffuse staining pattern. Only high-intensity foci observed in confocal optical sections were counted as aggregates. Previous studies demonstrated that DNAJB6 can condense, accumulates, and forms sarcoplasmic inclusions in skeletal muscle[1, 2], and that it colocalizes with aggregate-associated proteins such as Desmin in skeletal muscle from LGMD1D or myofibrillar myopathy patients[1, 3-5]. Based on these published studies, we used the term “aggregates” in the previous submission to describe the DNAJB6 puncta detected by immunofluorescence. However, because immunofluorescence alone may not be sufficient to establish biochemical insolubility as pointed out also by the other reviewers, we have revised the manuscript to use the more descriptive term “inclusions” to refer to these punctate structures by immunofluorescence. Please see the description of “*In vivo* muscle immunofluorescence staining and quantification” in the Methods section (page 17-18) for more details.
To enhance rigor, we increased the number of mice and quantified more than 1,500 muscle fibers per mouse across all related experiments (Figs. 1F, 5A, 7B-C, 8A-B) during this revision process. Importantly, the results remained consistent with our original findings. Moreover, in response to Reviewer 3’s suggestion (Point 7 and 9), we performed immunoblotting of soluble and insoluble fractions to detect DNAJB6, Desmin, and TDP43. Overall, immunoblotting from soluble and insoluble fraction showed consistent results with immunofluorescence data. Please also see our responses to Points 7 and 9 from R3 for more details.

8. The title indicates that Fbxl21 regulates "circadian proteostasis", however, the experiments presented do not demonstrate this - first, 2 timepoints is not enough "circadian" in my view, and second, most data do not indicate that the day-night different is eliminated, rather the PSTM mutant seem to be at an overall elevated levels (Figs 7-8) - thus not a "circadian" deficit by definition if the rhythm is still present.
Response: Thank you for your expert comment. We agree that two time points are not sufficient to capture the full profile of circadian rhythmicity. We therefore have revised the title to “FBXL21 regulates ***diurnal*** proteostasis and stress response via degradation of DNAJB6 and its client proteins” and made similar changes in relevant texts throughout the manuscript. Regarding the second point, we would like to clarify that we do not claim a complete loss of day–night differences. Rather, our data indicate that FBXL21 contributes to the diurnal

modulation of proteostasis capacity, manifested as time-of-day–dependent differences in baseline proteostasis and stress responsiveness. Whereas proteostatic markers such as FUS and TDP-43 showed diurnal rhythms in both WT and *Fbxl21* hypomorphic *Psttm* mice, their levels were elevated across the day–night cycle in *Psttm* mice, suggesting an altered diurnal setpoint or amplitude rather than complete arrhythmicity. This interpretation is consistent with extensive circadian studies in which mutation or knockout of clock components alters the oscillatory parameters (period, phase, amplitude, etc.) or gating of physiological responses without eliminating rhythmicity [6-11]. We have revised the title, Results, and Discussion to more precisely indicate that loss of FBXL21 alters diurnal proteostasis and stress resilience, rather than abolishing circadian rhythms altogether. For example, in Discussion (page 12): "Using timed exercise as a physiological stressor, we further found that FBXL21 contributes to the diurnal regulation of stress responses in skeletal muscle. In *Fbxl21* hypomorphic *Psttm* mice, levels of proteostatic markers such as G3BP1, FUS, and TDP-43 remained higher at ZT6 than at ZT18, yet elevated at both time points when compared to WT mice, suggesting an altered diurnal rhythm rather than complete loss of rhythmicity. These abnormalities were mitigated by the restoration of FBXL21 expression."

9. I'm curious - to Fbxl21 mutants have physiological muscle deficits that are or could be attributed to altered proteostasis? I was surprised this was not addressed, at least speculatively, in the MS.
Response: Thank you for your thoughtful comment. In our previous studies[6, 7], we showed that *Psttm* mice exhibit significant impairments in muscle performance, including reduced time to exhaustion, decreased running distance, and diminished grip strength, in part attributable to impaired circadian degradation of sarcomere protein substrates and subsequently sarcomere structural integrity[7]. However, given the severity and complexity of the muscle phenotypes observed, it is likely that additional FBXL21-targeted substrates also contribute to the phenotypes. Accumulating evidence indicates that dysregulation of proteostasis by impaired E3 ligase function can adversely affect muscle structure and function[12-14]. It is possible that the altered diurnal regulation of chaperone-dependent proteostasis reported herein is an important mechanism underlying the muscle dysfunctions in *Psttm* mice. Establishing a direct causal relationship, however, requires future studies. Reviewer 2 also made a similar comment (Point 5 below). Therefore, we have incorporated this integrated interpretation into the revised Discussion section (page 13, 2nd paragraph).

Reviewer 2 (R2): The manuscript by Lim and colleagues sought to expand our knowledge of circadian proteostasis via discovery of the Fbxl21 interactome and how this E3 ubiquitin ligase might degrade and regulate proteins that are important for the stress response system. The authors utilized an array of in vitro and in vivo approaches to target the Fbxl21 interactome. Firstly, Yeast hybrid screens and affinity pull down followed by mass spectrometry analysis was used to identify Fbxl21 targets. The studies were followed up by using Fbxl21 KO c2c12 cells to assess the Fbxl21, DNAJB6 and HSP70 interactions. The authors also used a Fbxl21 hypomorph mutant model (psttm) to study interactors in the context of stress response via an exercise stimulus. The authors also exposed C2C12 cells to heat stress to investigate stress granule resolution and the formation of protein aggregates under Fbxl21 KO conditions. A major finding from the current study was the regulation of DNAJB6 via Fbxl21 and how this altered turnover of client proteins, desmin and TDP-43. Detailed below are my comments.
1. The authors identified the changes in desmin aggregation as an outcome measure for a client protein DNAJB6 (Fig 5). It is worth noting in the discussion that E3 ligases MuRF1 and Trim32 in skeletal muscle have also been highlighted to regulate Desmin and change its solubility (PMID38283190; PMID22908310; PMID34179788). This is important given that E3 ligases are suggested have only specific substrates and little appreciation is given to the fact that E3s may work in tandem to degrade mutual substrates (PMID37955121).
Response: Thank you for the thoughtful comment. As the reviewer mentioned, ubiquitin-mediated proteasomal degradation of Desmin and other muscle proteins has been shown to be regulated by multiple E3 ligases[15-17]. While somewhat surprising, this is in fact consistent with the emerging view that ubiquitin-mediated degradation of a substrate protein can be temporally and contextually coordinated by functionally distinct E3 ligases[18]. My previous work - spanning more than 8 years - showed that two related E3 ligases, FBXL3 and FBXL21, perform

very different roles in degrading CRY proteins: while FBXL3 is a stronger E3 ligase responsible for CRY degradation in the nucleus, the relatively weaker E3 ligase FBXL21 is mainly localized in the cytoplasm, partially offsetting cytoplasmic FBXL3 activity[8, 9]. More specifically, MuRF1 (Trim63) and Trim32 have been shown to regulate Desmin solubility and turnover in skeletal muscle, particularly under catabolic or stress conditions[15, 16]. Furthermore, MYOZ1 and TCAP, two other FBXL21 substrates we previously reported, have also been shown to be ubiquitinated by MuRF1/MuRF2 (for MYOZ1)[19] and MuRF1 (for TCAP), respectively[20]. These results highlight FBXL21 and MuRF1 as E3 ligases targeting common substrates in skeletal muscle. Other examples of cooperating E3 ligases for the same targets include MDM2, COP1, CHIP, and Pirh2 for p53[21] and FBW7, HUWE1, SKP2, and TRIM32 for MYC[22]. Together, these findings are consistent with the notion that E3 ligases can act cooperatively or antagonistically on shared substrates rather than in isolation[18].

MuRF1 is mainly induced by catabolic and atrophy-associated signaling pathways, including FOXO and glucocorticoid activation, and functions primarily in structural protein turnover and muscle remodeling under stress conditions[23-25]. In comparison, the defining feature of FBXL21 is that it is a clock regulator (hypomorphic mutation leads to the short period phenotype in *Psttm*) while also serving as a clock-controlled E3 ligase that regulates ubiquitination of structure proteins and co-chaperone DNAJB6. Since previous reports showed circadian expression patterns of MuRF1 transcripts[26, 27], we performed a pilot experiment to determine whether MuRF1 protein expression exhibits circadian oscillation in the skeletal muscle (gastrocnemius) of WT mice. In contrast to the robust circadian expression of FBXL21 proteins (JTK_Cycle, $P<0.05$) as reported previously[7], MuRF1 protein levels showed a trend of increase in the active phase but did not reach the threshold for rhythmic expression in skeletal muscle (JTK_Cycle, $P=0.991$) (see the Response Letter figure, **RL-Fig. 1**). In addition, the *Psttm* mice showed significantly smaller muscle fiber diameters compared to WT[7]. This contrasts with canonical atrophy-associated E3 ligases such as MuRF1, whose deletion confers resistance to muscle atrophy and can in fact lead to increased muscle mass. Overall, while we fully agree that the coordinated degradation of substrate proteins by multiple E3 ligases, more specifically MuRF1 and FBXL21, is an important topic, future dedicated efforts are required to dissect their physiological roles and mechanistic relationship.

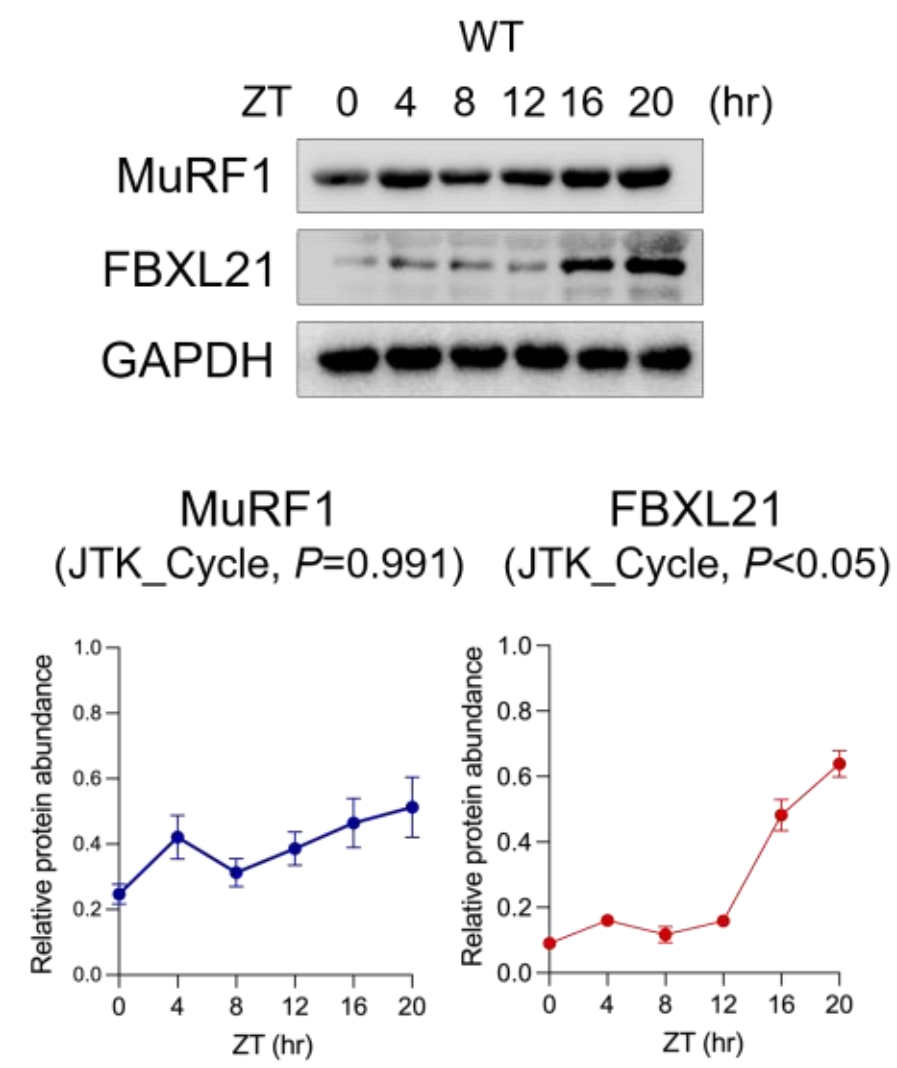

RL-Figure 1. MuRF1 and FBXL21 expression in skeletal muscle. MuRF1 and FBXL21 protein expression in skeletal muscle collected from WT mice at the indicated time points. Lower panels: quantification of MuRF1 and FBXL21 protein levels. Data are presented as mean ± SEM (n = 3 mice per time point). JTK analysis revealed that FBXL21 expression is rhythmic in WT mice (adjusted $^*P < 0.05$), whereas MuRF1 expression is not rhythmic (adjusted $P = 0.991$).

2. Did the *Psttm* mice display any exercise performance differences or the time to exhaustion between wild-type and *Psttm* mice?

Response: This comment is similar to the above Point 9 from R1. As mentioned above, in our previous study[7], we observed significant differences between WT and *Psttm* mice in exercise tolerance on treadmill. While WT mice showed a clear time-dependent pattern of running distance with a strong increase from ZT6 (inactive) to ZT18 (active), *Psttm* mice showed significantly reduced exercise performance at ZT18 and loss of the diurnal rhythm observed in WT, indicating an important role of FBXL21 in muscle function during the active phase.

3. Fig 1F, how were aggregates for DNAJB6 defined in skeletal muscle fibers? Given the size and composition of the lateral and medial gastrocnemius muscle, was the aggregation spread across these muscles or localized to a particular area? Did the authors account for regionality in the quantification? The use of more than 100 fibers as a threshold appears to be a low number, given the composition of the lateral and medial gastrocnemius muscle and its fiber density. The same aspect in this comment can be applied for Fig 5A about the quantification of desmin inclusion bodies across the tissue.

Response: Thank you for your thoughtful comments. As mentioned above in response to Point 7 from R1, we have now adopted the more generic term inclusions to replace aggregates, referring to well-demarcated, clear puncta or inclusion-like cytoplasmic structures distinguishable from the surrounding diffuse staining pattern. Regarding the distribution of inclusions, we did not observe any specific region of concentrated localization; rather, inclusions were observed throughout the gastrocnemius muscle. We also agree that the 100 fiber threshold is low, and we have increased to 1,500 fibers per mouse.

4. Given Fbxl21 function is reliant on SCF Complex interaction, is this complex altered in the Psttm mice? Is DNAJB6 targeted degradation dependent not only on Fbxl21 but also the SCF complex itself?

Response: Our previous study demonstrated that both WT and *Psttm* mutant FBXL21 proteins interact with CULLIN and SKP1 by BiFC (bimolecular fluorescence complementation) assays, although *Psttm* mutant protein stability is reduced compared to WT FBXL21[8]. As the reviewer correctly pointed out, F-box E3 ligases require SCF complex formation to transfer ubiquitin to target substrates. In a new experiment shown in **RL-Fig. 2**, we observed that F-box deletion (ΔFbxl21) abolished FBXL21 binding to SKP1 as well as its ubiquitination activity for DNAJB6. This new result has been added as Figure EV2E and described as follows on page 6: "To determine whether the FBXL21-mediated ubiquitination of DNAJB6b requires SCF complex assembly, we performed ubiquitination assays using an F-box deletion mutant of FBXL21 (ΔFbxl21). Deletion of the FBXL21 F-box abolished DNAJB6b ubiquitination (Fig. EV2E), indicating that SKP1 interaction and SCF complex assembly are required. Together, these results show that FBXL21 can promote the ubiquitination of DNAJB6 and its targeting to proteasomal degradation".

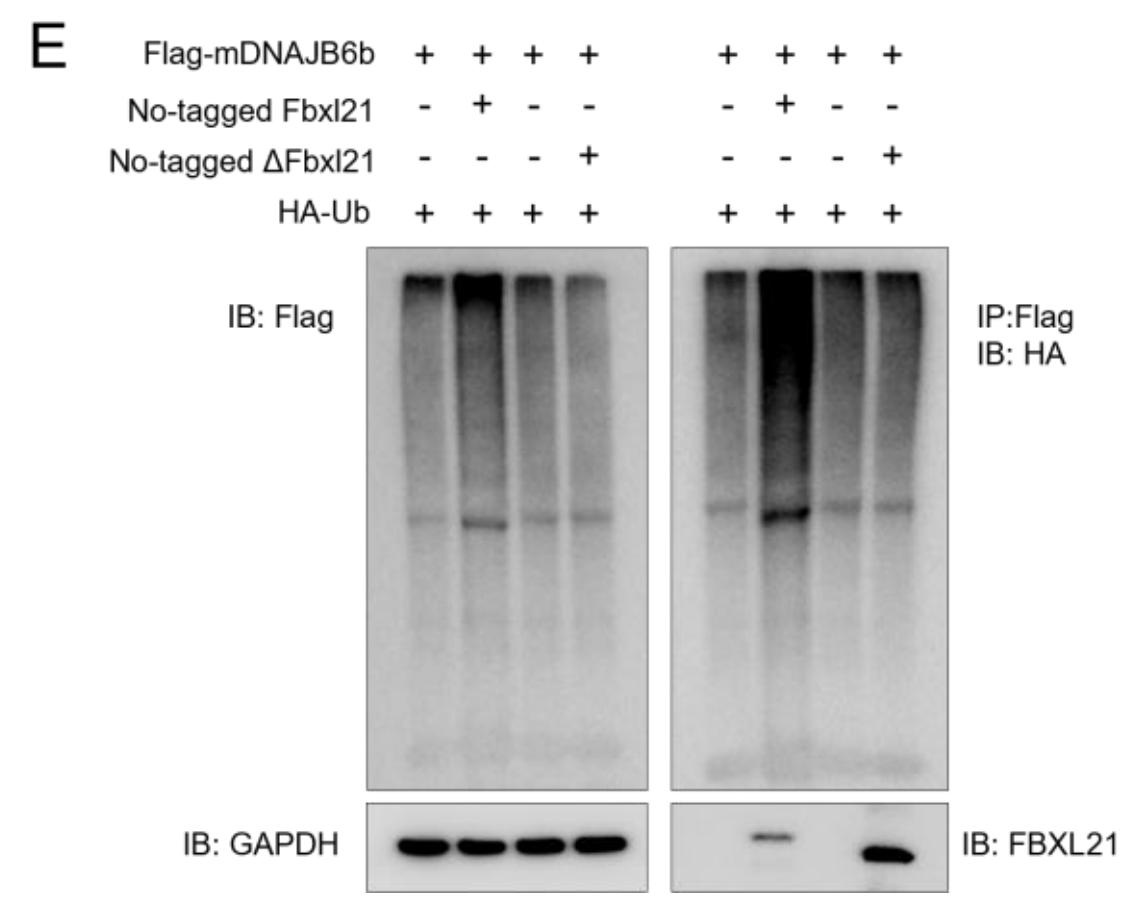

RL-Figure 2. ΔFbxl21 abolished the ability to ubiquitinate DNAJB6b. Flag-tagged Dnajb6b, untagged Fbxl21, untagged ΔFbxl21, and HA-tagged ubiquitin (HA-Ub) were co-transfected into 293T cells, followed by ubiquitination assays. Representative data from three independent experiments are shown.

5. It would be interesting to know if the changes in biochemical properties for stress granule formulation, TDP-43 and desmin aggregation leads to impaired skeletal muscle function in the current Psttm mice used? The authors highlight how these markers of proteotoxic stress contribute to other myopathies, yet discussion on skeletal muscle function in Psttm mice might benefit the reader too. Protein quality control can be compromised in skeletal muscle via E3 ligase knockdown, yet muscle mass can remain intact (PMID33658508).

Response: Thank you for this important comment. As we described in response to R1's Points 8–9, our previous study[7] demonstrated that *Psttm* mice exhibit impaired skeletal muscle performance, including a significant reduction of running distance and grip strength. As the reviewer correctly pointed out, muscle mass may remain largely normal under certain conditions of E3 ligase dysregulation[28]. Interestingly however, accumulating evidence suggests that muscle function can be compromised by dysregulated protein quality control independently of overt muscle wasting[14]. In *Psttm* mice with impaired FBXL21 activity, muscle fiber diameter was significantly shifted to smaller fiber sizes in *Psttm* compared to WT[7]. This contrasts with canonical atrophy-associated E3 ligases such as MuRF1, whose deletion confers resistance to muscle atrophy and can in fact lead to increased muscle mass. These observations suggest that FBXL21 does not function as a classical atrophy-related E3 ligase, but rather regulates diurnal proteostasis to influence skeletal muscle function.

A recent study has directly linked TDP-43 pathology to impaired muscle function and weakness[29], suggesting that aberrant TDP-43 accumulation is not merely a molecular marker but can drive functional deficits in muscle tissue and cause muscle degeneration. However, the mechanisms by which the accumulation of aggregation-prone proteins, including TDP-43, affects skeletal muscle function require further investigation. Moreover, the regulation of stress granule dynamics and TDP-43 accumulation in skeletal muscle under physiological

conditions, e.g., diurnal rhythms and exercise as a stressor, remains poorly characterized. Our findings reveal that exercise timing influences stress granule formation and TDP-43 accumulation, and that this diurnal regulation is disrupted in *Psttm* mice concomitant with significant muscle function impairments. Future studies will aim to establish a direct causal link between these cellular abnormalities and muscle performance deficits. We have added the following text in Discussion (page 13): "Previous studies showed that the *Fbxl21* hypomorphic *Psttm* mice exhibit impaired degradation of sarcomeric substrates and reduced muscle performance, including decreased running distance at nighttime and grip strength[7]. Of note, accumulating evidence indicates that dysregulated proteostasis and subsequent accumulation of aggregation-prone proteins, such as TDP-43 and Desmin, can adversely affect muscle structure and performance[12-14, 29-34]. In line with these findings, we show that disruption of the FBXL21–DNAJB6 axis in *Psttm* mice perturbs diurnal proteostasis and aggravates accumulation of aggregation-prone proteins, which may contribute to skeletal muscle dysfunction[7]."

<u>**Reviewer 3 (R3):**</u> The manuscript by Lim et al identifies a new interaction between FBXL21 and DNAJB6. Specifically, DNAJB6 is ubiquitinated by FBXL2, leading to its degradation; however, when DNAJB6 recognizes a client protein, this ubiquitination can then be transferred to the clients (Desmin and stress granule proteins) in a chaperone-dependent manner, akin to what is already established for proteins such as CHIP. Moreover, this occurs in circadian manner. The manuscript is conceptually interesting and proposes a novel mechanism for DNAJB6 mediated chaperone function but several issues remain.

1. The data in figure 1 demonstrates that DNAJB6 can associate with FBXL21 as found by unbiased IPs and yeast two hybrids. The increases in DNAJB6 in the nightime and with loss of FBXL21 could be due to a change in degradation but could also be due to increased gene expression. QPCR or other means of confirming that DNAJB6 isn't upregulated is necessary.

<u>Response:</u> Thank you for the suggestion. We performed qPCR using gastrocnemius tissues from WT and *Psttm* mice. The results showed that *Dnajb6* transcript levels did not differ significantly between WT and *Psttm* mice, suggesting that the accumulation of DNAJB6 in *Psttm* mice is primarily due to the impairment of FBXL21-mediated protein degradation rather than increased transcript expression (**RL-Fig. 3**). Interestingly, whereas DNAJB6 protein levels showed clear diurnal changes (Fig. 1D), the transcript levels were similar between the time points, further suggesting post-transcriptional regulation as a key regulatory layer for DNAJB6. This result has been added to Fig. S1D with the updated result description (page 5): "We next performed qPCR to examine *Dnajb6* mRNA levels in WT and *Psttm* mice (Fig. EV1D). *Dnajb6* mRNA levels were comparable between WT and *Psttm* mice and also between the two time points, suggesting that the diurnal patterns of DNAJB6 protein level and its increased abundance in *Psttm* mice are primarily due to post-transcriptional regulatory mechanisms". In Methods (pages 15-16), we have added a description of RNA extraction and real-time qPCR.

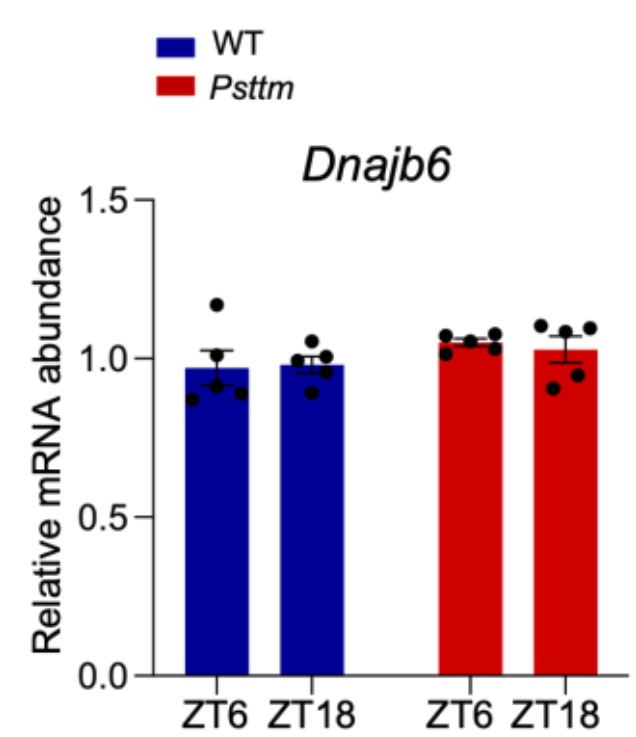

RL-Figure 3. mRNA levels of Dnajb6 in WT and *Psttm* mice. mRNA expression of *Dnajb6* in WT and *Psttm* mice. Data are presented as mean ± SEM (n =5/group). No mark indicates not statistically significant.

2. I am a bit confused by the claim in Figure 1D that there is reduced diurnal oscillation of DNAJB6 in the Psttm KO mice. the DNAJB6b quantified change look greater in the Psttm mice to me

<u>Response:</u> Apologies for the confusion. To clarify, DNAJB6a shows reduced diurnal oscillation in *Psttm* mice. In comparison, DNAJB6b retains its diurnal oscillation but at an exaggerated level in *Psttm* mice, as demonstrated by both immunoblotting and immunofluorescence assays. We have revised the description in the Results section (page 5): "Both DNAJB6a and DNAJB6b showed robust diurnal rhythms in WT (Fig. 1D). In *Psttm* mice, DNAJB6a levels were increased at ZT6, dampening the diurnal rhythm relative to WT; in comparison, DNAJB6b levels were similarly increased at both time points compared with WT (Fig. 1D)".

3. The immunofluorscense in skeletal muscle in figures 1F, 7 and 8 are difficult to interpret. Moreover, there is not methodology for how quantitation was performed, how mouse muscle was fixed and sectioned. Relative

fluorscence intensity does not seem appropriate (is it for an individual fiber? In the case of 1F, how is a DNAJB6 aggregate defined. Is DNAJB6 now insoluble via fractionation, ubiquitinated. The diurnal change is not very convincing via IF.

Response: Thank you for the thoughtful comments. The comment regarding immunofluorescence methodology and quantification procedures is similar to Point 7 from R1 and Point 3 from R2 – please see the responses above. Regarding the diurnal effect, we agree that immunofluorescence alone is likely not sensitive enough to fully capture the rhythmic amplitude or assess protein insolubility. To address this point, we prepared soluble and insoluble fractions of skeletal muscle lysates from WT and *Psttm* mice, followed by immunoblot analysis (**RL-Fig. 4**). Our new results show that DNAJB6a and DNAJB6b protein levels exhibited diurnal rhythms in WT mice in both fractions. Specifically, for the new Fig. EV1E (page 5): "Next, soluble and insoluble fractions were prepared from gastrocnemius muscles from WT and *Psttm* mice followed by immunoblot analysis. In the soluble fractions, both DNAJB6a and DNAJB6b levels displayed diurnal rhythms in WT and *Psttm* mice, with higher levels generally observed in *Psttm* (Fig. EV1E). In the insoluble fractions, the diurnal rhythm of DNAJB6a in WT mice was abolished in *Psttm* mice. In comparison, insoluble DNAJB6b exhibited diurnal rhythms in both WT and *Psttm* mice, with exaggerated levels detected in *Psttm* mice." We also added the method of "detergent-soluble and insoluble fractionation" on page 16.

In addition, we added the fractionation description in the Methods section (page 16).

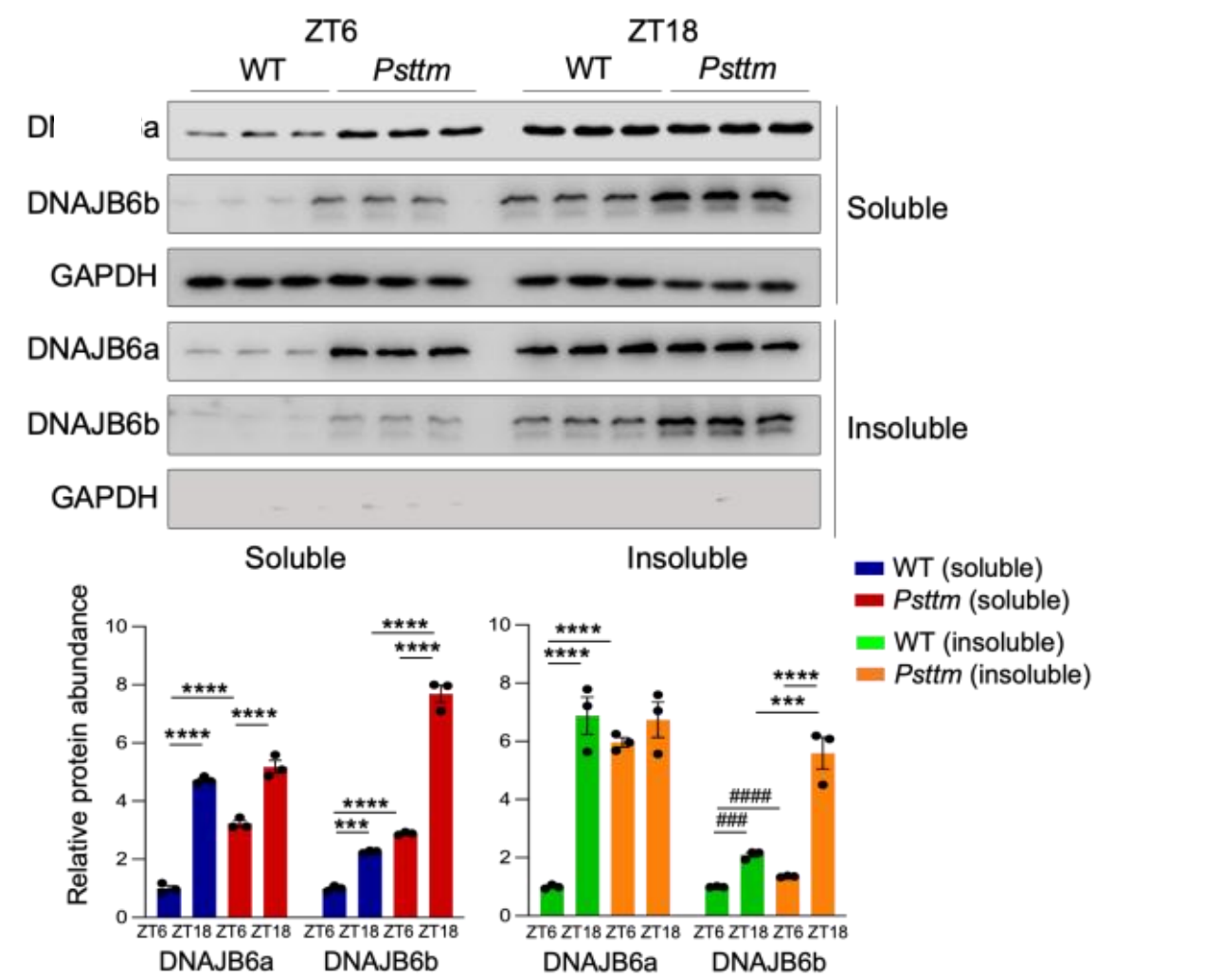

RL-Figure 4. Immunoblot of DNAJB6 in soluble and insoluble fractions from WT and *Psttm* mice. GAPDH was used as a positive control for the soluble fraction. Data are presented as mean ± SEM (n = 3 mice/group/time point). ***P = 0.0002 and ****P < 0.0001; Two-way ANOVA with Tukey's multiple comparisons test, comparing between WT and *Psttm* mice or between time points. $^{###}P$ = 0.0002 and $^{####}P$ < 0.0001; Unpaired Student's t-test indicates significant differences between specific comparisons.

4. Figure 2 D and E would benefit from some molecular weight markers.

Response: We have added molecular weight markers to Figures 2D and 2E. Thank you.

5. Figure 3 is an important figure. Does Fbxl21 associate with HSP70 independently of DNAJB6 or as a complex. Figure 1A would suggest it can occur independently. Does Figure 3B and 3C have the levels of HSP70 and FBXl21 in the reciprocal treatments. Figure 3e should show that FBxl21 is similarly expressed in the control and chip SiRNA

Response: We thank the reviewer for these valuable comments. To determine whether FBXL21 associates with HSP70 independently of DNAJB6 or as part of a complex, we performed a new co-immunoprecipitation experiment. We transfected 293T cells with V5-tagged HSP70 and Flag-tagged FBXL21, with or without DNAJB6 siRNA (**RL-Fig. 5**). Subsequently, we pulled down the protein complex using Flag beads. The results showed that FBXL21 interacts with HSP70 even in the absence of DNAJB6, indicating that FBXL21 can bind HSP70 independently of DNAJB6. These data have been added as the new Figure 3B (on page 6). "DNAJB6 knockdown did not affect FBXL21-HSP70 interaction, suggesting that FBXL21 can interact with HSP70 independently of DNAJB6 (Fig. 3B)".

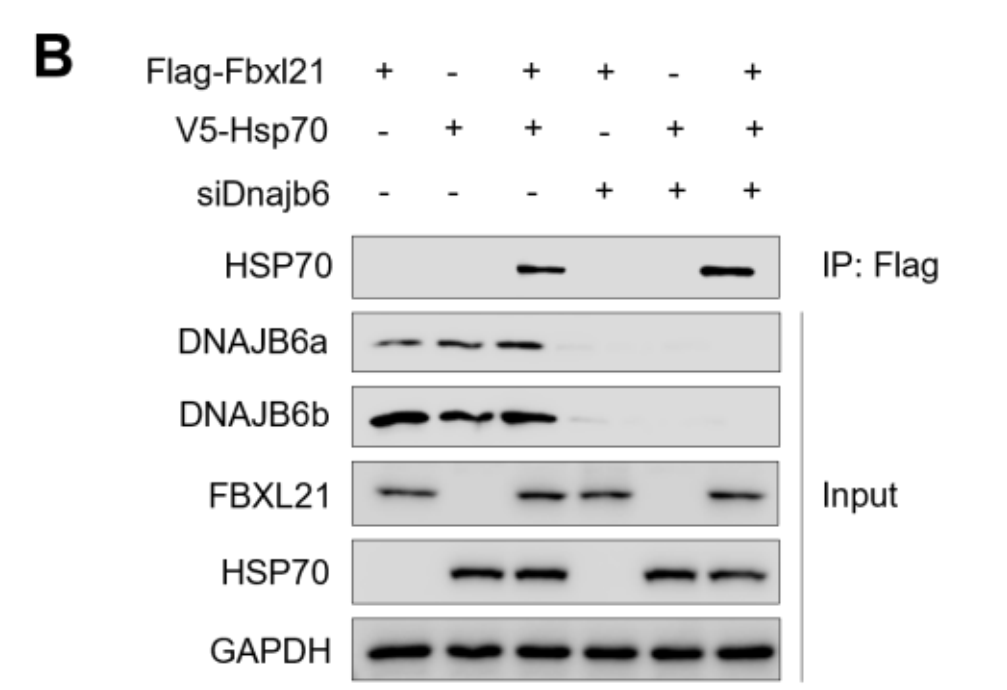

RL-Figure 5. FBXL21 interacts with HSP70 independently of DNAJB6. Flag-tagged Fbxl21 and V5-tagged Hsp70 were co-transfected into 293T cells with or without Dnajb6 siRNA, followed by co-immunoprecipitation. Representative data from three independent experiments are shown.

In addition, we updated Figures 3C, 3D and 3F to show that the levels of HSP70 and FBXL21 in the reciprocal IPs are comparable across treatments, and that FBXL21 expression remains similar in control and CHIP siRNA conditions.

6. In Figure 4, are LGMDD1 mutants not ubiquitinated? In addition, Figure 4C needs to show the input of DNAJB6 levels and the expression levels of HSP70 in the tissue.

Response: In response to the reviewer's question, we performed ubiquitination assays to examine whether LGMDD1 mutants are ubiquitinated by FBXL21 (**RL-Fig. 6**). We found that the ubiquitination levels of two LGMDD1 mutants by FBXL21 were markedly reduced compared to WT DNAJB6b. This suggests that disease-associated mutations in DNAJB6 impair FBXL21-mediated ubiquitination, potentially affecting its turnover and chaperone activity. These data have been added as the new Figure 4C and described in the Results section (page 7) as follows: "Moreover, both mutants showed markedly reduced ubiquitination by FBXL21 (Fig. 4C)."

Regarding the previous Figure 4C (now Figure 4D), we have included the input levels of DNAJB6 to provide a clear reference for the immunoprecipitation results in Figure 4D (**RL-Fig. 7**). The input levels of HSP70 in the tissues were already included in the original figure. Thank you for these constructive comments.

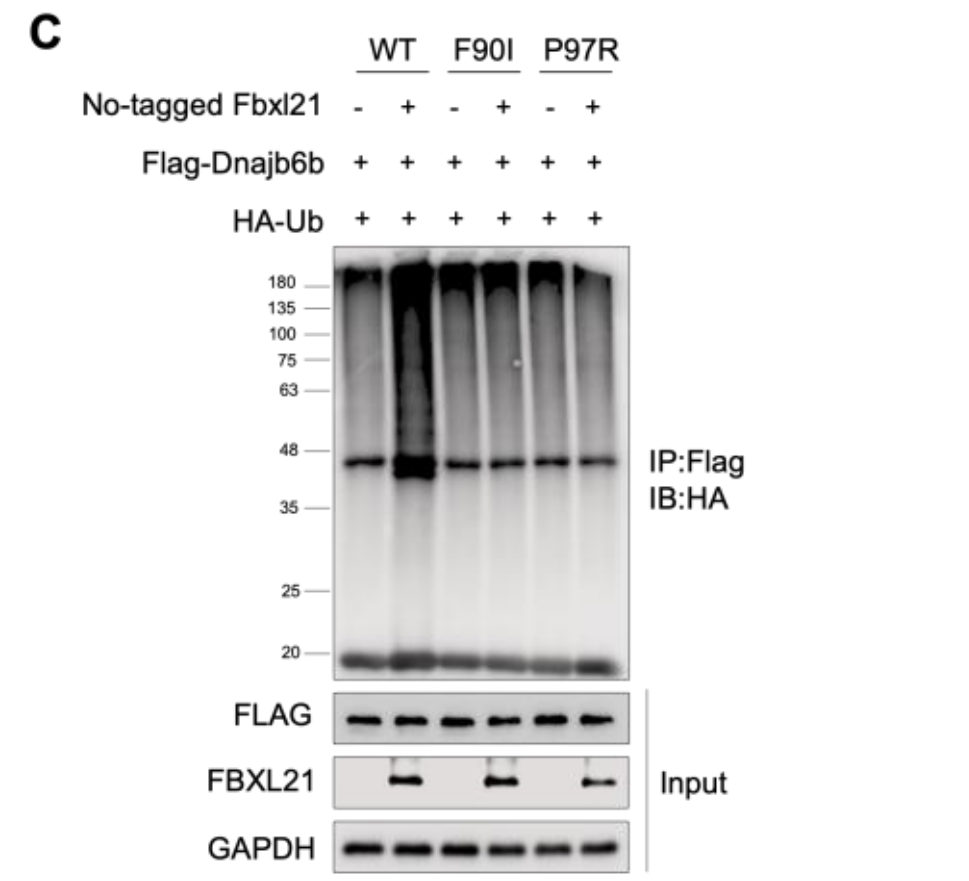

RL-Figure 6. Ubiquitination assays of DNAJB6 mutants. Flag-tagged Dnajb6 and Dnajb6 mutants, untagged Fbxl21, and HA-tagged ubiquitin (HA-Ub) were co-transfected into 293T cells, followed by ubiquitination assays. Representative data from three independent experiments are shown.

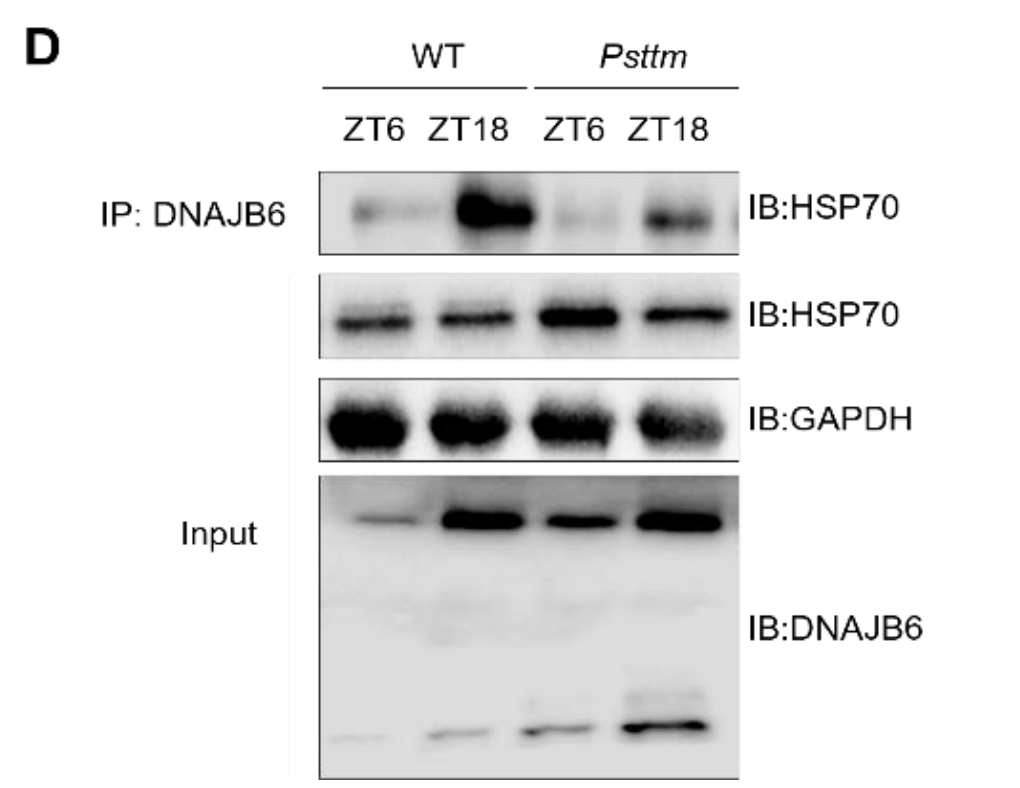

RL-Figure 7. New Figure 4D (updated DNAJB6 input). Co-immunoprecipitation of DNAJB6/HSP70 from gastrocnemius tissues of WT and *Psttm* mice at ZT6 and ZT18, using an anti-DNAJB6 antibody.

7. Figure 5A has similar issues regarding IF in skeletal muscle; these look oversaturated, and how do we define and aggregate. The IF images look like every fiber has an aggregate but the quantiation just says 2-6% of fibers. Does the desmin or DNAJB6 become insoluble. Figure 5B, it is difficult to know what is being quantitated. puncta/fluorescence intensity. Figure 5E is important and needs to show blots for all the transfected proteins to confirm they are equal across conditions.

Response: We appreciate these thoughtful comments similar to Point 7 from R1 and Point 3 from R2. For Figure 5A, the initial exposure settings were chosen to clearly visualize Desmin- and DNAJB6-positive inclusions and illustrate their morphology. We take the reviewer's concern regarding potential oversaturation to heart and have replaced the previous images with unsaturated ones that are still suitable for quantification. As mentioned in response to similar comments above, we have increased the N numbers and quantified more than 1,500 fibers per mouse. Specifically, approximately 500 myofibers per section were analyzed (in total, more than 1,500 fibers), and the percentage of fibers containing discrete Desmin- or DNAJB6-positive inclusions was calculated. Desmin and DNAJB6-positive inclusions (%) were between 5-8%. We have clarified this in the revised figure legend and Methods (pages 17-18).

As already mentioned in response to the comments from the other reviewers, Desmin aggregation is a well-characterized feature of desminopathy and related myofibrillar myopathies, where detergent-insoluble Desmin-positive aggregates are commonly observed[1, 3, 35 4]. Based on this established pathology, we initially used the term "aggregates" to describe the punctate Desmin-positive structures detected by immunofluorescence. However, as the reviewer correctly noted, immunofluorescence alone may not be sufficient to establish biochemical insolubility. We have therefore revised the manuscript to use more generic terminology such as "Desmin-positive inclusions" or "puncta". Future studies will further characterize their biochemical properties.

To support the immunofluorescence observations, we performed biochemical fractionation of gastrocnemius muscle lysates into soluble and detergent-insoluble fractions, followed by immunoblotting for DNAJB6 and Desmin. Both proteins were significantly increased in the insoluble fraction from *Psttm* mice compared with WT at both time points. For DNAJB6, please see the response to Point 3 from R2 (**RL-Fig. 4**); for Desmin, please see **RL-Fig. 8**. We have added the following sentence on page 8 to describe this new result: "Consistent with this observation, Desmin accumulation in both soluble and insoluble fractions was significantly elevated in *Psttm* skeletal muscle at both time points (Fig. EV3D)." For Figure 5B, we have clarified in the Methods that quantification was performed as mean fluorescence intensity per area, using uniform thresholding parameters applied across all groups. Together, these biochemical data complement IF results to strengthen the interpretation of altered protein solubility.

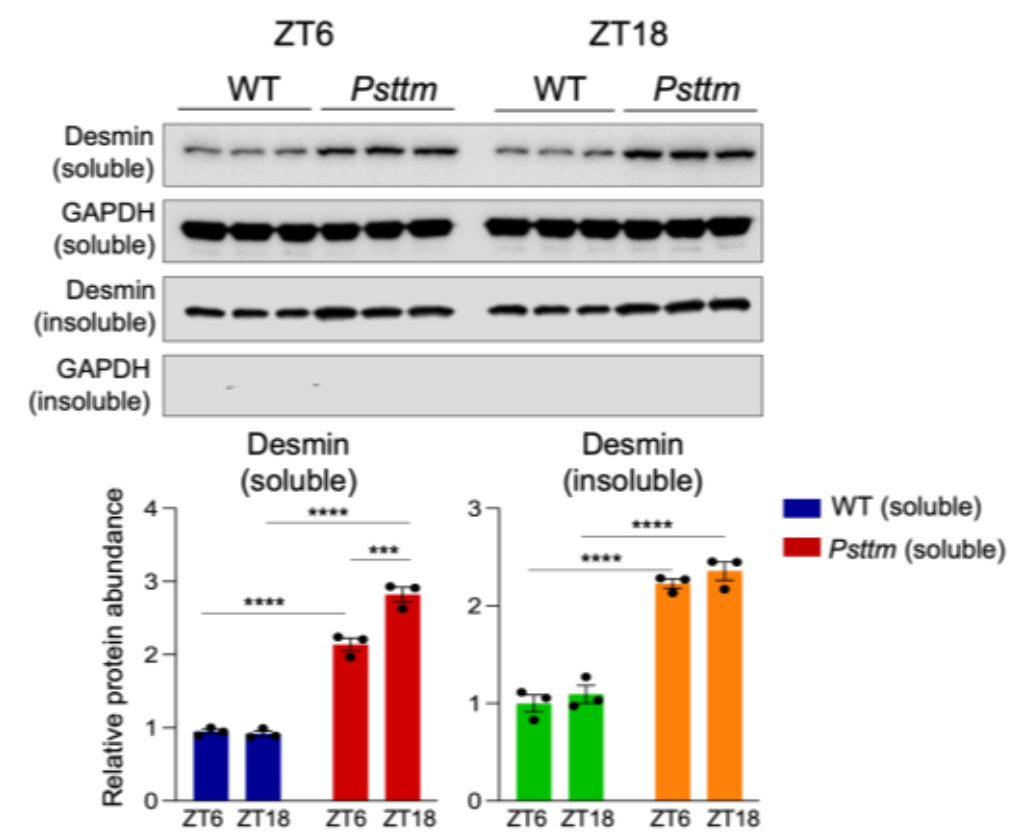

RL-Figure 8. Immunoblot of Desmin in soluble and insoluble fractions of WT and *Psttm* mice. GAPDH was used as a positive control for the soluble fraction. Data are presented as mean ± SEM (n = 3). ***P = 0.0001 and ****P < 0.0001; Two-way ANOVA with Tukey's multiple comparisons test, comparing between WT and *Psttm* mice or between time points.

For Figure 5E, we have included immunoblots for all transfected proteins in this panel to confirm comparable expression levels across conditions (**RL-Fig. 9**). Lysates were run and probed together, and we observed consistent results. All inputs have been added to ensure that the observed differences in input levels are consistent in other transfected groups.

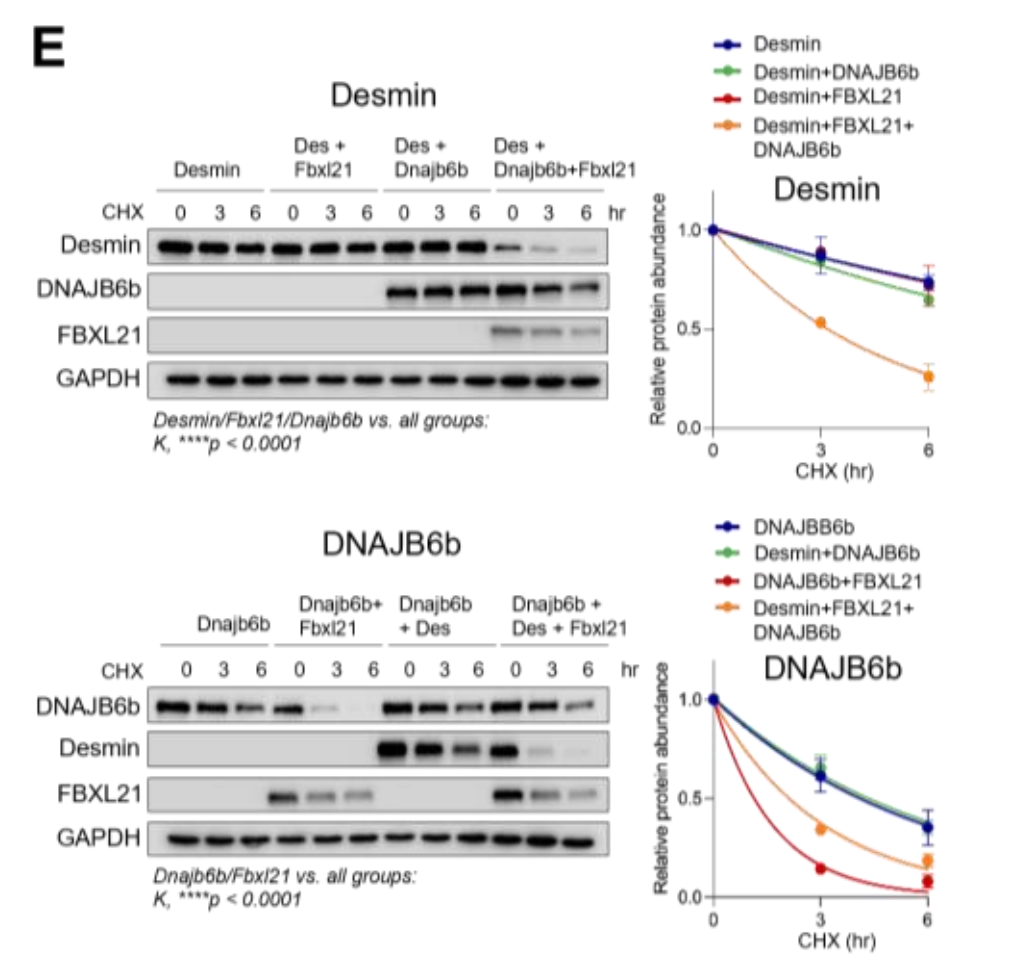

RL-Figure 9. CHX chase assays assessing Desmin and DNAJB6b stability in 293T cells co-transfected with Flag-tagged Desmin, Fbxl21, and Dnajb6b. Left panel (Desmin half-lives): Desmin, 14.20 h; Desmin/Fbxl21, 14.75 h; Desmin/Dnajb6b, 11.63 h; Desmin/Fbxl21/Dnajb6b, 3.33 h (K, Desmin/Fbxl21/Dnajb6b significantly different from all other groups, ****P < 0.0001). Right panel (Dnajb6b half-lives): Dnajb6b, 4.35 h; Dnajb6b/Fbxl21, 1.26 h; Dnajb6b/Desmin, 4.57 h; Dnajb6b/Desmin/Fbxl21, 2.35 h (K, Dnajb6b/Fbxl21 significantly different from all other groups, ****P < 0.0001). Data are presented as mean ± SEM (n = 4).

8. Figure 6 is not really showing dynamics, just formation of stress granules. and formation of TDP-43 cytosolic puncta. Not entirely clear what they are quantifying. Figure S5A no gapdh Figure S5C polyQ aggregates form on their own and not with heat shock. I am not certain what phenomenon is being measured. The control that is needed is a non-expanded polyQ.

Response: We thank the reviewer for the thoughtful comments. First, we agree that Figure 6 does not directly assess stress granule or TDP-43 dynamics, but rather the formation of G3BP1-positive stress granules and cytosolic TDP-43 puncta under defined stress conditions. To avoid overinterpretation, we have revised the text throughout the manuscript to state "formation" rather than "dynamics." Regarding quantification, we have clarified in the revised figure legend and Methods that the analysis was performed by counting the number of G3BP1-positive stress granules and TDP-43–positive cytosolic puncta per cell from more than 100 cells, using uniform thresholding criteria applied across all groups. We have added the following sentence in Methods (page 17): "For *in vitro* quantification, we counted more than 100 cells per group with uniform thresholding criteria applied across all groups."

For Figure S5A, GAPDH loading controls were included in the original Figure S5A (now Fig. EV3C) to ensure proper normalization. Regarding polyQ aggregation in Figure S5C (now Fig. EV3F), the expanded polyQ constructs are widely used as a functional readout of DNAJB6 chaperone activity[36-40]. In our study, aggregation of expanded polyQ was moderate in the cultured cells under basal conditions used; therefore, we included heat-shock conditions to more easily assess whether DNAJB6 chaperone function is compromised in *Fbxl21* KO C2C12 cells. We quantified the relative aggregate area (%) according to the published method[41-43]. Specifically, the total aggregate area (%) per cell was measured and normalized to the corresponding total cell area, then

multiplied by 100 to obtain the percentage of aggregate burden. This quantification method is now described in Methods (pages 16-17) and mentioned in figure legends for clarity.

In response to the reviewer's suggestion, we performed the same experiment using a non-expanded polyQ construct (Q23htt-EGFP). The results showed that Q23htt displayed diffuse cytoplasmic localization and did not form detectable puncta under either basal or heat shock conditions in both WT and *Fbxl21* KO C2C12 cells, confirming that puncta formation is specific to the expanded polyQ construct (**RL-Fig.10**). These data have been added as the new Figure EV3F, and the manuscript has been revised accordingly. Specifically, on page 8-9: "As a negative control, we used the non-expanded polyQ construct GFP-polyQ23 and found no aggregation in both control and *Fbxl21* KO C2C12 cells, confirming that the observed aggregation was specific to polyQ-expanded proteins (Fig. EV3F)." On pages 15-16, the Methods section is updated.

We thank the reviewer again for the helpful comments.

F

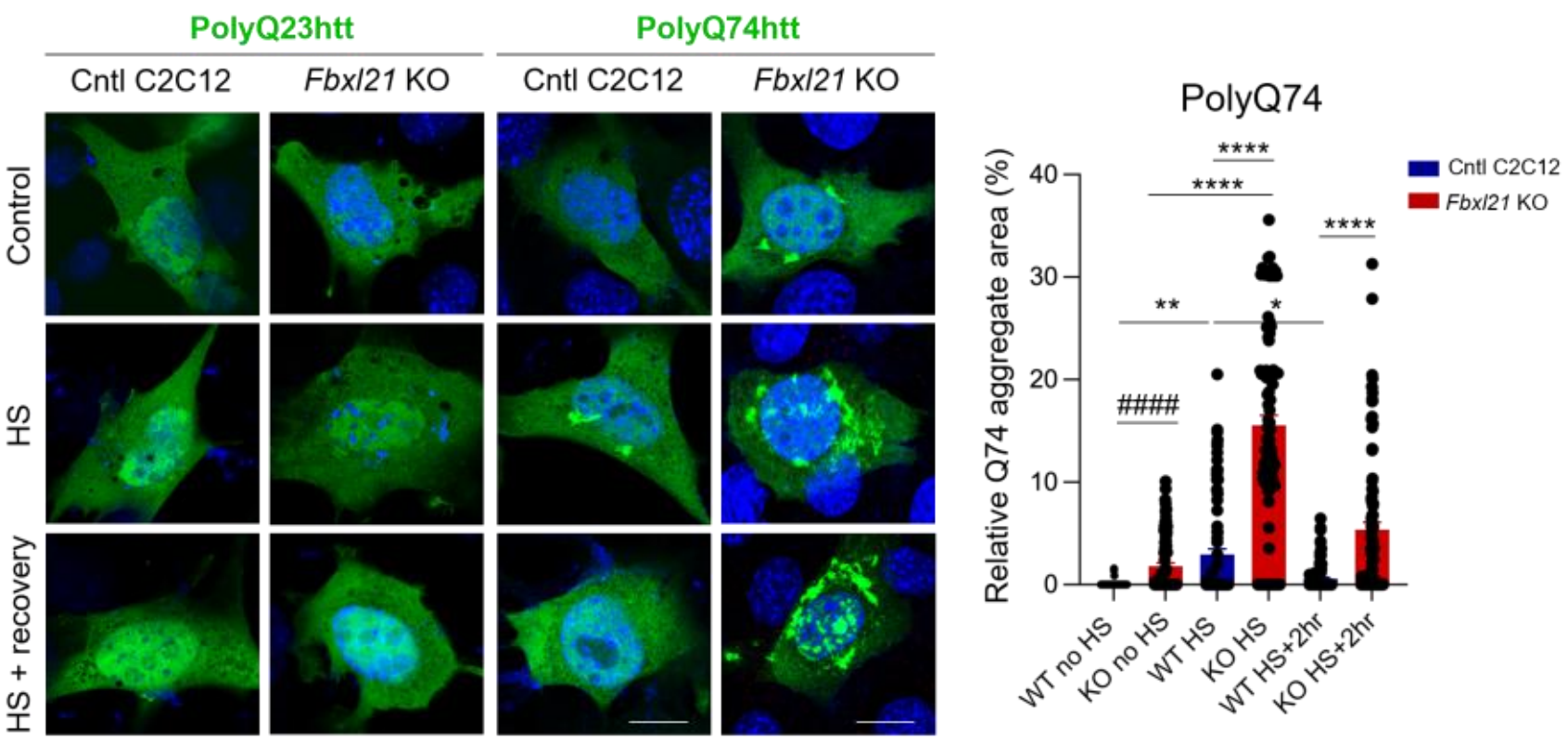

RL-Figure 10. Confocal microscopy analysis in Cntl and *Fbxl21* KO C2C12 cells expressing polyQ23htt-EGFP and polyQ74htt-EGFP. PolyQ23htt-EGFP was used as a negative control, and no aggregates were detected in the groups. The relative Q74 aggregate area (%) was quantified using ImageJ. Data are presented as mean ± SEM (n=100 cells/group). **P* = 0.0253 (WT HS vs. WT HS recovery), ***P* = 0.0023 (WT no HS vs. WT HS), and *****P* < 0.0001; One-way ANOVA showing significant differences between groups. ####*P* < 0.0001; Unpaired Student's *t*-test.

9. Figure 7 and 8 Suffer from similar issues. The reliance of IF and unclear quantitation based on relative fluorescent intensity. the paper calls these stress granules but not sure what they are quantitating. I am not certain how one can be certain these are stress granules as a stress granule is reversible and we don't know if these are aggregates or stress granules. Immunoblotting showing insolubility or other markers would be helpful. Quantitation of loss of TDP-43 from the nuclear may be relevant or demonstration of cryptic splicing.

Response: We agree that immunofluorescence alone is not sufficient to definitively distinguish stress granules from insoluble aggregates. Although there is literature describing "formation of stress granules in the tissues" based on G3BP1 as a stress granule marker [44-46], some other literature just reported it as "G3BP1 expression or G3BP1 accumulation." [47] To avoid overinterpretation, we have revised the manuscript—particularly in Figures 7 and 8—to describe the observed changes as alterations in stress granule marker expression rather than definitive stress granule structures or dynamics.

Regarding quantification, please see the response to R1's Point 7.

To provide biochemical support for the immunofluorescence findings, we performed detergent-based fractionation of gastrocnemius muscle lysates from WT and *Psttm* mice at ZT6 and ZT18, followed by immunoblotting for TDP-43. Soluble TDP-43 accumulation showed a similar pattern observed by immunofluorescence staining results, whereas insoluble TDP-43 was detected only after acute exercise. Insoluble TDP-43 levels were higher in *Psttm* mice compared with WT mice (**RL-Fig. 11**). Interestingly, muscle-specific expression of *Fbxl21* significantly reduced the insoluble accumulation of TDP43. These new data (added as the new Fig. 8C) are described in the Results section (page 11).

To examine whether cytoplasmic accumulation of TDP-43 in *Psttm* mice was accompanied by nuclear depletion, we quantified nuclear TDP-43 immunofluorescence intensity using a previously reported method[48]. Briefly, three random fields were selected from gastrocnemius cross-sections of WT and *Psttm* mice. DAPI staining was used to define nuclear boundaries, which were overlaid with TDP-43 images, and the background outside the nuclei was removed using the "Clear Outside" function in ImageJ. Mean fluorescence intensity within the nuclear area

was then quantified. In total, more than 1,500 fibers per mouse per group were analyzed. Interestingly, nuclear TDP-43 intensity was not reduced in *Psttm* mice compared with WT under either control or exercise conditions (**RL-Fig. 12**). Consistent with our data in Fig. 8A–C showing increased total TDP-43 levels in *Psttm* muscle, these results suggest that the elevated sarcoplasmic TDP-43 signal is unlikely to result from nuclear depletion. Together, these additional biochemical data and clarifications strengthen the interpretation that *Psttm* mice exhibit exaggerated TDP43 accumulation in the skeletal muscle under both basal and exercise conditions and restoration of FBXL21 expression rescues this defect.

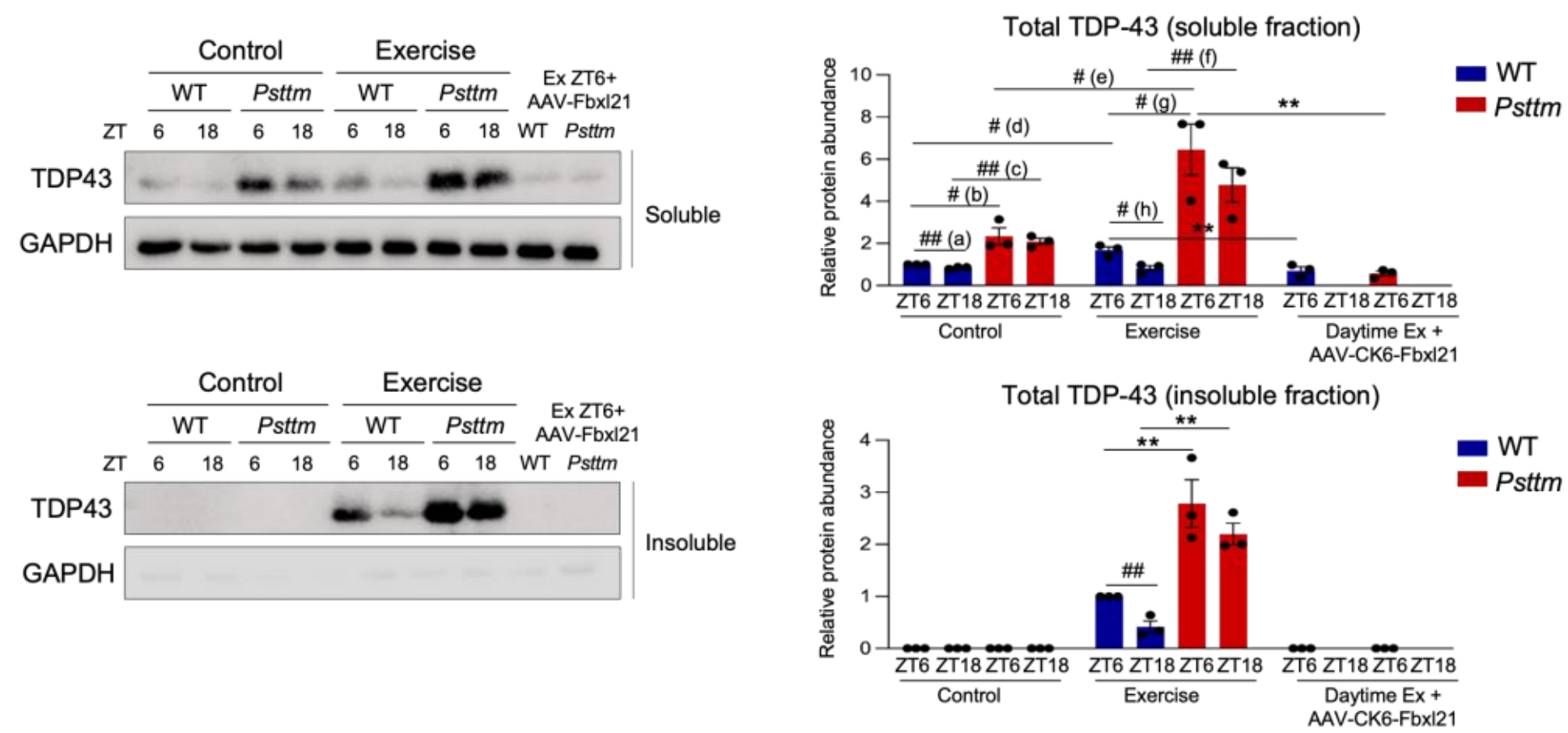

RL-Figure 11. TDP-43 protein levels in the soluble and insoluble fractions in WT and *Psttm* mice subjected to acute exercise. Data are presented as mean ± SEM (n = 3 mice/group/time point). For total TDP-43 (soluble fraction), $^{**}P$ = 0.0066 (Daytime Ex WT vs. Daytime Ex WT+ AAV-CK6-Fbxl21) and $^{**}P$ = 0.0066 (Daytime Ex *Psttm* vs. Daytime Ex *Psttm* + AAV-CK6-Fbxl21*)*; Two-way ANOVA indicates statistical differences between groups. $^{\#\#(a)}P$ =0.0063, $^{\#(b)}P$ =0.0289, $^{\#\#(c)}P$ =0.0012, $^{\#(d)}P$ =0.0141, $^{\#(e)}P$ =0.0324, $^{\#\#(f)}P$ =0.0087, $^{\#(g)}P$ =0.0175, and $^{\#(h)}P$ =0.0157; Student's *t*-test indicates significant differences between specific comparisons. For total TDP-43 (insoluble fraction), $^{**}P$ = 0.0051 (exercise WT ZT6 vs. exercise *Psttm* ZT6), $^{**}P$ = 0.0052 (exercise WT ZT18 vs. exercise *Psttm* ZT18); One-way ANOVA indicates statistical differences between groups. $^{\#\#}P$ = 0.0067; Unpaired Student's *t*-test indicates significant differences between specific comparisons. N numbers indicate biological replicates for each sample group and representative data from 3 independent experiments are shown.

We sincerely thank the reviewers for insightful and constructive comments. In response, we have performed new experiments and analyses, clarified key points, and strengthened the presentation of our data in the revised manuscript. We believe these revisions markedly improve the overall quality and rigor of the manuscript. We hope that you now find the manuscript suitable for publication in *EMBO Reports*.

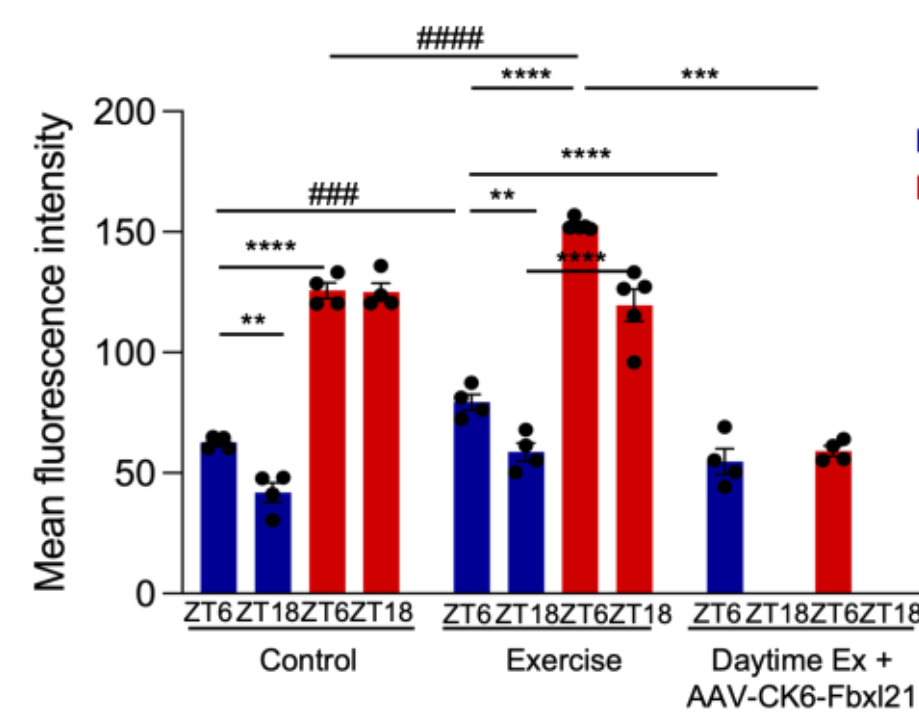

RL-Figure 12. TDP43 nuclear fluorescence intensity in WT and *Psttm* mice. Data are presented as mean ± SEM (n = 4-5 mice per group). $^{**}p < 0.01$, $^{***}p < 0.001$, and $^{****}p < 0.0001$; Two-way ANOVA indicates statistical differences between groups. $^{\#\#\#}p < 0.001$ and $^{\#\#\#\#}p < 0.0001$; Student's *t*-test indicates significant differences between specific comparisons.

References

1. Sarparanta J, Jonson PH, Golzio C, et al. Mutations affecting the cytoplasmic functions of the co-chaperone DNAJB6 cause limb-girdle muscular dystrophy. Nat Genet. 2012;44(4):450-455, S451-452.

2. Qian FY, Guo YD, Zu J, et al. A novel recessive mutation affecting DNAJB6a causes myofibrillar myopathy. Acta neuropathologica communications. 2021;9(1):23.

3. Bertini E, Bosman C, Ricci E, et al. Neuromyopathy and restrictive cardiomyopathy with accumulation of intermediate filaments: a clinical, morphological and biochemical study. Acta Neuropathol. 1991;81(6):632-640.

4. Bertini E, Salviati G, Apollo F, et al. Reducing body myopathy and desmin storage in skeletal muscle: morphological and biochemical findings. Acta Neuropathol. 1994;87(1):106-112.

5. Joanne P, Chourbagi O, Hourde C, et al. Viral-mediated expression of desmin mutants to create mouse models of myofibrillar myopathy. Skeletal muscle. 2013;3(1):4.

6. Lim JY, Kim E, Douglas CM, et al. The circadian E3 ligase FBXL21 regulates myoblast differentiation and sarcomere architecture via MYOZ1 ubiquitination and NFAT signaling. PLoS Genet. 2022;18(12):e1010574.

7. Wirianto M, Yang J, Kim E, et al. The GSK-3beta-FBXL21 Axis Contributes to Circadian TCAP Degradation and Skeletal Muscle Function. Cell reports. 2020;32(11):108140.

8. Yoo SH, Mohawk JA, Siepka SM, et al. Competing E3 Ubiquitin Ligases Govern Circadian Periodicity by Degradation of CRY in Nucleus and Cytoplasm. Cell. 2013;152(5):1091-1105.

9. Siepka SM, Yoo SH, Park J, et al. Circadian mutant Overtime reveals F-box protein FBXL3 regulation of cryptochrome and period gene expression. Cell. 2007;129(5):1011-1023.

10. Takahashi JS. Transcriptional architecture of the mammalian circadian clock. Nat Rev Genet. 2017;18(3):164-179.

11. Takahashi JS, Hong HK, Ko CH, et al. The genetics of mammalian circadian order and disorder: implications for physiology and disease. Nat Rev Genet. 2008;9(10):764-775.

12. Shemer S. Mechanisms of protein degradation in atrophying muscles: what have we learned during the past decade? The Journal of biological chemistry. 2026:111229.

13. Hughes DC, Baehr LM, Waddell DS, et al. Ubiquitin Ligases in Longevity and Aging Skeletal Muscle. International journal of molecular sciences. 2022;23(14).

14. Fernando R, Drescher C, Nowotny K, et al. Impaired proteostasis during skeletal muscle aging. Free radical biology & medicine. 2019;132:58-66.

15. Lord SO, Dawson PWJ, Chunthorng-Orn J, et al. Uncovering the mechanisms of MuRF1-induced ubiquitylation and revealing similarities with MuRF2 and MuRF3. Biochem Biophys Rep. 2024;37:101636.

16. Cohen S, Zhai B, Gygi SP, et al. Ubiquitylation by Trim32 causes coupled loss of desmin, Z-bands, and thin filaments in muscle atrophy. J Cell Biol. 2012;198(4):575-589.

17. Baehr LM, Hughes DC, Lynch SA, et al. Identification of the MuRF1 Skeletal Muscle Ubiquitylome Through Quantitative Proteomics. Function (Oxf). 2021;2(4):zqab029.

18. Hughes DC, Goodman CA, Baehr LM, et al. A critical discussion on the relationship between E3 ubiquitin ligases, protein degradation, and skeletal muscle wasting: it's not that simple. Am J Physiol Cell Physiol. 2023;325(6):C1567-C1582.

19. Moriscot AS, Baptista IL, Bogomolovas J, et al. MuRF1 is a muscle fiber-type II associated factor and together with MuRF2 regulates type-II fiber trophicity and maintenance. J Struct Biol. 2010;170(2):344-353.

20. Polge C, Cabantous S, Deval C, et al. A muscle-specific MuRF1-E2 network requires stabilization of MuRF1-E2 complexes by telethonin, a newly identified substrate. J Cachexia Sarcopenia Muscle. 2018;9(1):129-145.

21. Sane S, Rezvani K. Essential Roles of E3 Ubiquitin Ligases in p53 Regulation. International journal of molecular sciences. 2017;18(2).

22. Farrell AS, Sears RC. MYC degradation. Cold Spring Harbor perspectives in medicine. 2014;4(3).

23. Waddell DS, Baehr LM, van den Brandt J, et al. The glucocorticoid receptor and FOXO1 synergistically activate the skeletal muscle atrophy-associated MuRF1 gene. Am J Physiol Endocrinol Metab. 2008;295(4):E785-797.

24. Bodine SC, Latres E, Baumhueter S, et al. Identification of ubiquitin ligases required for skeletal muscle atrophy. Science. 2001;294(5547):1704-1708.

25. Shimizu H, Langenbacher AD, Huang J, et al. The Calcineurin-FoxO-MuRF1 signaling pathway regulates myofibril integrity in cardiomyocytes. Elife. 2017;6.

26. McCarthy JJ, Andrews JL, McDearmon EL, et al. Identification of the circadian transcriptome in adult mouse skeletal muscle. Physiol Genomics. 2007;31(1):86-95.

27. Zhang H, Liang J, Chen N. Do not neglect the role of circadian rhythm in muscle atrophy. Ageing Res Rev. 2020;63:101155.

28. Hunt LC, Schadeberg B, Stover J, et al. Antagonistic control of myofiber size and muscle protein quality control by the ubiquitin ligase UBR4 during aging. Nature communications. 2021;12(1):1418.

29. Lynch EM, Pittman S, Daw J, et al. Seeding-competent TDP-43 persists in human patient and mouse muscle. Science translational medicine. 2024;16(775):eadp5730.

30. Mori F, Tada M, Kon T, et al. Phosphorylated TDP-43 aggregates in skeletal and cardiac muscle are a marker of myogenic degeneration in amyotrophic lateral sclerosis and various conditions. Acta neuropathologica communications. 2019;7(1):165.

31. Weihl CC, Temiz P, Miller SE, et al. TDP-43 accumulation in inclusion body myopathy muscle suggests a common pathogenic mechanism with frontotemporal dementia. J Neurol Neurosurg Psychiatry. 2008;79(10):1186-1189.

32. Salajegheh M, Pinkus JL, Taylor JP, et al. Sarcoplasmic redistribution of nuclear TDP-43 in inclusion body myositis. Muscle Nerve. 2009;40(1):19-31.

33. Kedia N, Arhzaouy K, Pittman SK, et al. Desmin forms toxic, seeding-competent amyloid aggregates that persist in muscle fibers. Proc Natl Acad Sci U S A. 2019;116(34):16835-16840.

34. van Spaendonck-Zwarts KY, van Hessem L, Jongbloed JD, et al. Desmin-related myopathy. Clin Genet. 2011;80(4):354-366.

35. Clemen CS, Herrmann H, Strelkov SV, et al. Desminopathies: pathology and mechanisms. Acta Neuropathol. 2013;125(1):47-75.

36. Mansson C, Kakkar V, Monsellier E, et al. DNAJB6 is a peptide-binding chaperone which can suppress amyloid fibrillation of polyglutamine peptides at substoichiometric molar ratios. Cell Stress Chaperones. 2014;19(2):227-239.

37. Gillis J, Schipper-Krom S, Juenemann K, et al. The DNAJB6 and DNAJB8 protein chaperones prevent intracellular aggregation of polyglutamine peptides. The Journal of biological chemistry. 2013;288(24):17225-17237.

38. Bason M, Meister-Broekema M, Alberts N, et al. Astrocytic expression of the chaperone DNAJB6 results in non-cell autonomous protection in Huntington's disease. Neurobiology of disease. 2019;124:108-117.

39. Thiruvalluvan A, de Mattos EP, Brunsting JF, et al. DNAJB6, a Key Factor in Neuronal Sensitivity to Amyloidogenesis. Mol Cell. 2020;78(2):346-358 e349.

40. Joshi BS, Youssef SA, Bron R, et al. DNAJB6b-enriched small extracellular vesicles decrease polyglutamine aggregation in in vitro and in vivo models of Huntington disease. iScience. 2021;24(11):103282.

41. Klickstein JA, Mukkavalli S, Raman M. AggreCount: an unbiased image analysis tool for identifying and quantifying cellular aggregates in a spatially defined manner. The Journal of biological chemistry. 2020;295(51):17672-17683.

42. Korner M, Muller P, Das H, et al. p97/VCP is required for piecemeal autophagy of aggresomes. Nature communications. 2025;16(1):4243.

43. Fisch D, Pfleiderer MM, Anastasakou E, et al. PIM1 controls GBP1 activity to limit self-damage and to guard against pathogen infection. Science. 2023;382(6666):eadg2253.

44. Yang X, Li G, Lou P, et al. Excessive nucleic acid R-loops induce mitochondria-dependent epithelial cell necroptosis and drive spontaneous intestinal inflammation. Proc Natl Acad Sci U S A. 2024;121(1):e2307395120.

45. Zhou Y, Yang Z, Wang Y, et al. Stress granule assembly impairs macrophage efferocytosis to aggravate allergic rhinitis in mice. Nature communications. 2025;16(1):5610.

46. Sanchez, II, Nguyen TB, England WE, et al. Huntington's disease mice and human brain tissue exhibit increased G3BP1 granules and TDP43 mislocalization. The Journal of clinical investigation. 2021;131(12).

47. Herman AB, Silva Afonso M, Kelemen SE, et al. Regulation of Stress Granule Formation by Inflammation, Vascular Injury, and Atherosclerosis. Arteriosclerosis, thrombosis, and vascular biology. 2019;39(10):2014-2027.

48. Sen B, Xie Z, Thomas MD, et al. Nuclear actin structure regulates chromatin accessibility. Nature communications. 2024;15(1):4095.

Dear Dr. Yoo

Thank you for the submission of your revised manuscript to EMBO reports. I sincerely apologize for the unusual delay in handling your manuscript. Only one of the three former referees has provided a report on the revised manuscript. Given that referee #3 had a number of important concerns, I have decided to ask an independent, additional expert in the field to evaluate your response to the concerns from referee #3. The advisor's comments are copied below (referee #4).

As you will see, both experts are both very positive about the study and request only minor changes to clarify text and figures.

From the editorial side, there are also a few things that we need before we can proceed with the official acceptance of your study.

- The manuscript sections should be in the following order: Title page - Abstract & Keywords - Introduction - Results - Discussion - Methods - Data Availability - Acknowledgments - Disclosure Statement & Competing Interests - References - Figure Legends - (Main Tables with legends if applicable) - Expanded View Figure Legends.

- Please note that "Materials and Methods" should be "Methods."

- Please note that the Conflict of Interest section should be called "Disclosure and Competing Interests Statement".

- The references need to be alphabetical, not numerical; et al needs to be used after 10 author names.

- Please provide a complete author checklist, which you can download from our author guidelines. Please insert information in the checklist that is also reflected in the manuscript. The completed author checklist will also be part of the Review Process File.

- The information on funding provided in the manuscrpit text and the online submission system must be congruent. It is the latter that will be transferred to PubMed. Here we note that the Welch Foundation (AU-2127-20220331) is missing in the system as a separate file; it needs to be added via the More Funders option (not in the Comments box). Moreover, there is a discrepancy between NIH grant number R01A1158694 provided in the manuscript and R01AI158694 entered in the system.

- Figure callouts: Fig. S1H is not a correct callout; please check whether this refers to Figure EV1H? The callout to Appendix Table EV1 in the Data availability section should be changed to Dataset EV1, if I am not mistaken (see below).

- Please provide Table EV1 in .xls format and call it Dataset EV1. Please provide the legend in a separate tab of the .xls file. Please update the nomenclature in all instances (file name, legend, callouts)

- Data availability: please deposit the mass spectrometry data on an appropriate public repository in addition to providing the results in Dataset EV1 and provide a link to the dataset.

- Please provide the Reagents and Tools table in Word format and upload it as Reagents table file type.

- Figure 7, please acknowledge BioRender in the methods section (only) as follows:
Graphics:
(some of the... OR Figure #... OR synopsis) Graphics were created with BioRender.com.

- The resolution of the submitted figures is too low for blot and microscopy images. This reduction in resolution is commonly caused by converting original 16-bit TIFF files to RGB format for publication. While this is not inherently problematic, it can raise concerns about image integrity for critical readers.

To avoid any misunderstanding and to meet EMBO Press standards, we kindly ask that you:

* Resubmit the complete figure set at the captured original data resolution.

* Apply the same resolution standards to the blot source data files, which are also currently below the required quality threshold. Please upload the blot source data files as .tiff or pdf. Please do not use PowerPoint.

- Please address the following points in the figure legends and please highlight the changes:
* Please indicate the statistical test used for data analysis in the legends of figures 2B, C; 3C, D, F, G; 4B, 5D, E
* Please note that the white arrow heads are not defined in the legend of figure 6C. This needs to be rectified.
* Figure 6C, 8A, EV4: please provide a scale bar for the enlarged images.

* Figure EV3F: please define the scale bars and add scale bars for the PolyQ23htt images.

- Western blots: please add molecular weight markers in the panels.

- As a standard procedure, we edit title and abstract of all manuscripts to make them more accessible to our general readership. Please find my suggestions below my signature for your review.

- Finally, EMBO Reports papers are accompanied online by
A) a short (1-2 sentences) summary of the findings and their significance,
B) 2-3 bullet points highlighting key results and
C) a schematic summary figure that provides a sketch of the major findings (not a data image).
Please provide the summary figure as a separate file in PNG or JPG format at a size of 550x300 pixels (width x height). Please note that the size is rather small and that text needs to be readable at the final size. Please send us this information along with the revised manuscript.

We look forward to seeing a final version of your manuscript as soon as possible.

With kind regards,

Martina Rembold, PhD
Senior Editor
EMBO reports

==================

Referee #2:

The authors have done an excellent job addressing my concerns and comments from the initial submission. The manuscript is significantly improved and will be a welcome addition to the field.

Referee #4:

I have read the manuscript and the comments from reviewer 3. All in all, I think that the authors addressed the comments raised by reviewer 3 very well and did a great job in revising the manuscript.

There are still 3 minor issues that should still be addressed.

1) Regarding point 3 (reviewer 3): Since the lysis buffer used for the soluble-insoluble fractionation for Fig. EV1E is very mild (0.5% triton), the authors should include that specification directly in the figure/graph and manuscript text to avoid confusion with insolubility studies of e.g., polyQ aggregates which are 2% SDS insoluble.

2) Regarding point 9 (reviewer 3): I appreciate the clarification of the image quantification. Given that not stress granules per se but rather protein expression based on IF is quantified, the last sentence on page 10 of the section "FBXL21 regulates exercise timing-dependent stress response in skeletal muscle" should be corrected. That is, the phrasing "aggravating stress granule accumulation" should be removed since this was not quantified and to avoid misleading.

3) Additionally, and since I am working with polyQ aggregation, I think that the claim/statement of figure EV3F is pre-mature. It is correct that DNAJB6 is a very potent chaperone that acts on polyQ aggregation, however, there are also other factors that influence polyQ aggregation in cells. Given the observation here, the fact that Fbxl21 KO leads to more polyQ aggregates can be due to DNAJB6 functioning, but there is no direct proof shown for this experiment.
I would therefore suggest in the sentence ("We next examined whether FBXL21 depletion affects DNAJB6 activity to suppress polyQ aggregation.") to remove "DNAJB6 activity to suppress" since there is no causal proof given for that.

================

FBXL21 regulates diurnal proteostasis in skeletal muscle by targeting DNAJB6 and client proteins

OR

FBXL21 regulates diurnal proteostasis and stress responses in muscle by targeting DNAJB6 and client proteins

Circadian regulation of proteostasis, a key determinant of muscle health, remains poorly understood. Here, we identify DNAJB6, an Hsp40 (DnaJ) co-chaperone, as a substrate of the circadian E3 ligase FBXL21. FBXL21 mediates the ubiquitination-dependent proteasomal degradation of both DNAJB6 and its client proteins including Desmin. In contrast, myopathy-causing mutations of DNAJB6 confer resistance to FBXL21-directed degradation. Fbxl21 KO C2C12 cells display aberrant accumulation of Desmin, and show aggravated cytoplasmic accumulation of TDP-43, another DNAJB6 client protein, in response to heat shock. Under timed exercise as a physiological stressor, WT mice display robust diurnal rhythms in the levels of stress granule markers (G3BP1 and FUS) and of TDP-43 as a function of exercise timing. In contrast, the Fbxl21 hypomorph Psttm mutant mice show elevated expression of these proteins without exercise, which is exacerbated under exercise-induced stress conditions. Importantly, these abnormalities are rescued by skeletal muscle-specific FBXL21 expression. Our study elucidates a novel diurnal regulatory mechanism of skeletal muscle proteostasis via FBXL21 as a chaperone-linked E3 ligase, highlighting the FBXL21-DNAJB6 axis as a potential therapeutic target for myopathies.

Point-by-point response to peer review comments

We thank the expert reviewers for their thoughtful comments on our revised manuscript. Point-by-point responses to the remaining comments are as follows.

Referee #2: The authors have done an excellent job addressing my concerns and comments from the initial submission. The manuscript is significantly improved and will be a welcome addition to the field.

Response: We thank the reviewer for the positive assessment and for the many constructive comments during the first round which helped us strengthen the manuscript significantly.

Referee #4: I have read the manuscript and the comments from reviewer 3. All in all, I think that the authors addressed the comments raised by reviewer 3 very well and did a great job in revising the manuscript.

There are still 3 minor issues that should still be addressed.

Response: We thank this expert reviewer for taking the time to review the overall revision response and providing the positive assessment and additional constructive comments.

1. Since the lysis buffer used for the soluble–insoluble fractionation for Fig. EV1E is very mild (0.5% Triton), the authors should include that specification directly in the figure/graph and manuscript text to avoid confusion with insolubility studies of e.g. polyQ aggregates which are 2% SDS insoluble.

Response: We agree and thank the reviewer for raising this important point. The reviewer is correct that our soluble/insoluble fractionation used a mild lysis buffer (0.5% Triton X-100), which differs from the stringent 2% SDS conditions typically used to assess the detergent-resistant insolubility of polyQ aggregates. To prevent any confusion, we have specified the condition in figures, figure legends and the main text. Specifically, we now label the fractions as “0.5% Triton X-100 soluble or insoluble” in the Figs. 8C, EV1E, and EV3D. We have also added “0.5% Triton X-100” in the figure legends on pages 31, 32, and 33. And on page 5, “Next, 0.5% Triton X-100 soluble and insoluble fractions were prepared from gastrocnemius muscles from WT and *Psttm* mice followed by immunoblot analysis.”

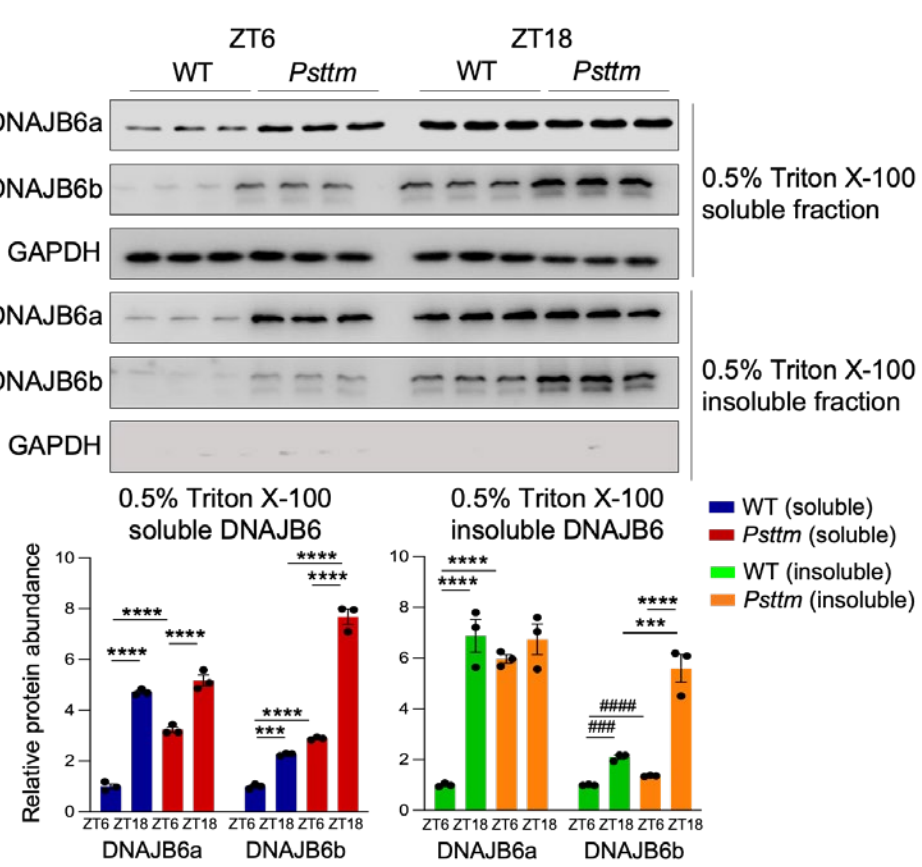

Figure EV1E. Immunoblot of DNAJB6 in 0.5% Triton X-100 soluble and insoluble fractions from WT and *Psttm* mice.

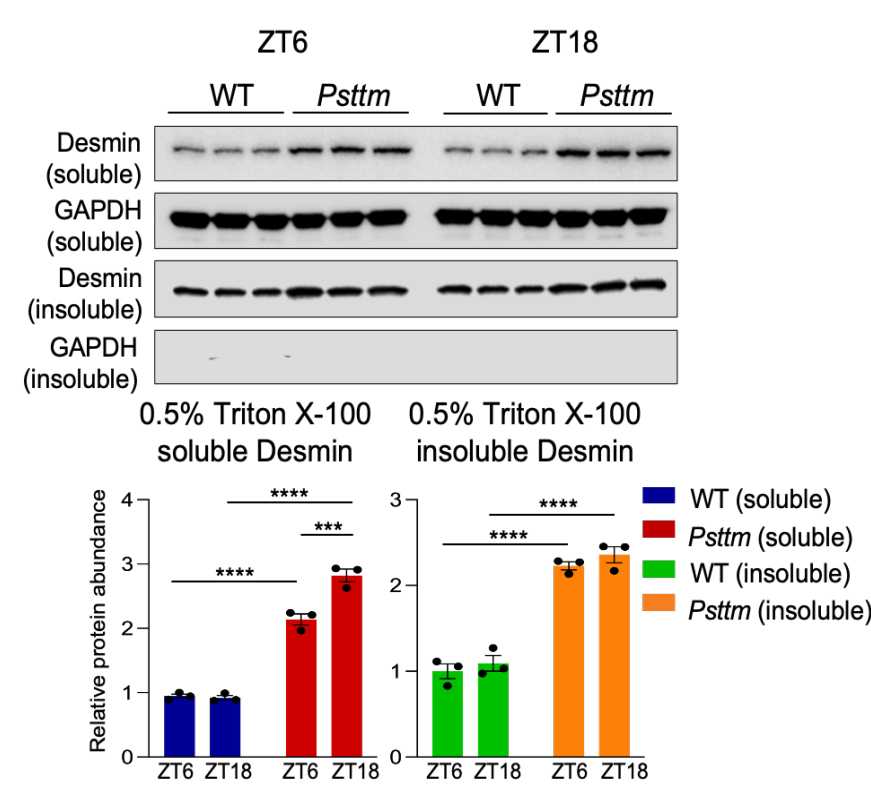

Figure EV3D. Immunoblot of Desmin in 0.5% Triton X-100 soluble and insoluble fractions from WT and *Psttm* mice.

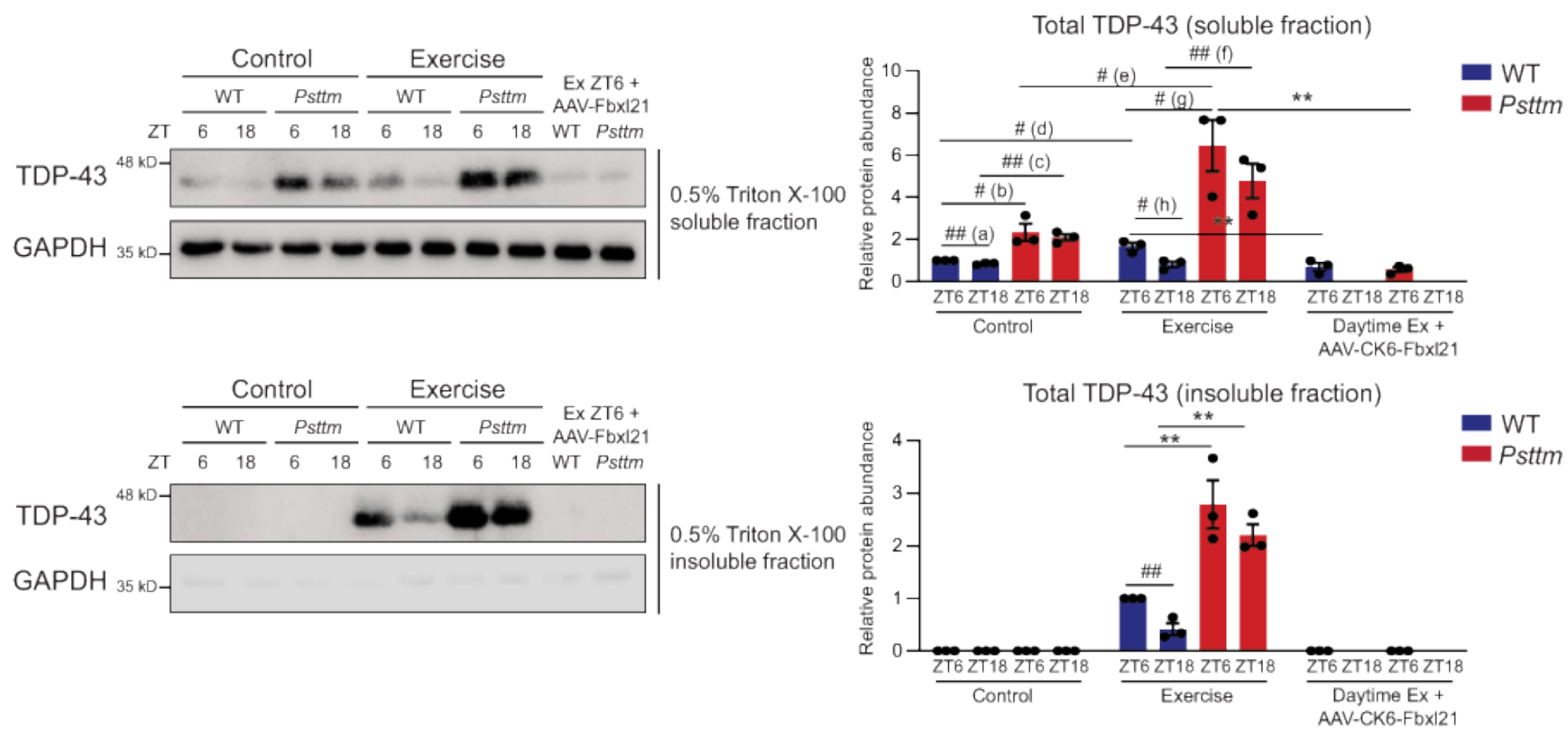

Figure. 8C. TDP-43 protein levels in the 0.5% Triton X-100 soluble and insoluble fractions from WT and *Psttm* mice subjected to acute exercise.

2. Given that not stress granules per se but rather protein expression based on IF is quantified, the last sentence on page 10 of the section "FBXL21 regulates exercise timing-dependent stress response in skeletal muscle" should be corrected. The phrasing "aggravating stress granule accumulation" should be removed since this was not quantified and to avoid misleading.

Response: We thank the reviewer for this expert comment. We agree that our immunofluorescence analysis directly quantified stress granule marker protein levels rather than stress granule number or area *per se*. We have removed "aggravating stress granule accumulation" and replaced it with "may affect stress granule marker expression in the muscles." On page 11, the complete sentence is now as follows: "These results suggest that the FBXL21-DNAJB6 axis in *Psttm* mice is impaired in client protein degradation, which may contribute to the increased G3BP1 and FUS levels." (changes highlighted)

3. The claim/statement of Figure EV3F is premature. DNAJB6 is a very potent chaperone that acts on polyQ aggregation; however, other factors also influence polyQ aggregation in cells. The finding that Fbxl21 KO leads to more polyQ aggregates can be due to DNAJB6 functioning, but there is no direct proof for this. I would suggest removing "DNAJB6 activity to suppress" from the sentence "We next examined whether FBXL21 depletion affects DNAJB6 activity to suppress polyQ aggregation," since there is no causal proof given.

Response: We thank the reviewer for this expert point. We agree that the increased polyQ aggregation in *Fbxl21* KO C2C12 cells may not result solely from the altered DNAJB6 activity in this experiment, given that other factors can be involved. As the reviewer suggested, we have removed "DNAJB6 activity to suppress" from the sentence; the sentence is now as follows (page 9): "We next examined whether FBXL21 depletion affects polyQ aggregation."

Thank you again for the constructive critiques. We hope you find our manuscript now suitable for publication at *EMBO Reports*.

2nd Revision - Editorial Decision **20th Aug 2026**

Seung-Hee Yoo
The University of Texas Health Science Center at Houston
Department of Biochemistry and Molecular Biology
Houston 77030
United States

Dear Dr. Yoo,

I am very pleased to accept your manuscript for publication in the next available issue of EMBO reports. Thank you for your contribution to our journal.

Your manuscript will be processed for publication by EMBO Press. It will be copy edited and you will receive page proofs prior to publication. Please note that you will be contacted by Springer Nature Author Services to complete licensing and payment information.

You may qualify for financial assistance for your publication charges - either via a Springer Nature fully open access agreement or an EMBO initiative. Check your eligibility: https://link.springer.com/journal/44319/how-to-publish-with-us

Should you be planning a Press Release on your article, please notify the production team via this form in order to coordinate publication and release dates: https://forms.cloud.microsoft/e/eH4A8JVP6g

If you have any questions, please do not hesitate to contact the Editorial Office. Thank you for your contribution to EMBO Reports.

Yours sincerely,

Martina Rembold, PhD
Senior Editor
EMBO reports

>>> Please note that it is EMBO Reports policy for the transcript of the editorial process (containing referee reports and your response letter) to be published as an online supplement to each paper. If you do NOT want this, you will need to inform the Editorial Office via email immediately. More information is available here: https://link.springer.com/partners/embo-press/editorial-policies#Peer%20review

EMBO Press Author Checklist

Corresponding Author Name: Seung-Hee Yoo	
Journal Submitted to: EMBO Reports	
Manuscript Number: EMBOR-2025-63032V	

USEFUL LINKS FOR COMPLETING THIS FORM
The EMBO Journal - Author Guidelines
EMBO Reports - Author Guidelines
Molecular Systems Biology - Author Guidelines
EMBO Molecular Medicine - Author Guidelines

Reporting Checklist for Life Science Articles (updated January
This checklist is adapted from Materials Design Analysis Reporting (MDAR) Checklist for Authors. MDAR establishes a minimum set of requirements in transparent reporting in the life sciences (see Statement of Task: 10.31222/osf.io/9sm4x). Please follow the journal's guidelines in preparing your
Please note that a copy of this checklist will be published alongside your article.

Abridged guidelines for figures
1. Data
The data shown in figures should satisfy the following conditions:
- the data were obtained and processed according to the field's best practice and are presented to reflect the results of the experiments in an accurate and unbiased manner.
- ideally, figure panels should include only measurements that are directly comparable to each other and obtained with the same assay.
- plots include clearly labeled error bars for independent experiments and sample sizes. Unless justified, error bars should not be shown for technical
- if n<5, the individual data points from each experiment should be plotted. Any statistical test employed should be justified.
- Source Data should be included to report the data underlying figures according to the guidelines set out in the authorship guidelines on Data

2. Captions
Each figure caption should contain the following information, for each panel where they are relevant:
- a specification of the experimental system investigated (eg cell line, species name).
- the assay(s) and method(s) used to carry out the reported observations and measurements.
- an explicit mention of the biological and chemical entity(ies) that are being measured.
- an explicit mention of the biological and chemical entity(ies) that are altered/varied/perturbed in a controlled manner.
- the exact sample size (n) for each experimental group/condition, given as a number, not a range;
- a description of the sample collection allowing the reader to understand whether the samples represent technical or biological replicates (including how many animals, litters, cultures, etc.).
- a statement of how many times the experiment shown was independently replicated in the laboratory.
- definitions of statistical methods and measures:
  - common tests, such as t-test (please specify whether paired vs. unpaired), simple χ2 tests, Wilcoxon and Mann-Whitney tests, can be unambiguously identified by name only, but more complex techniques should be described in the methods section;
  - are tests one-sided or two-sided?
  - are there adjustments for multiple comparisons?
  - exact statistical test results, e.g., P values = x but not P values < x;
  - definition of 'center values' as median or average;
  - definition of error bars as s.d. or s.e.m.

Please complete ALL of the questions below.
Select "Not Applicable" only when the requested information is not relevant for your study.

Materials

Newly Created Materials	Information included in the manuscript?	In which section is the information available? (Reagents and Tools Table, Materials and Methods, Figures, Data Availability Section)
New materials and reagents need to be available; do any restrictions apply?	Yes	Reagents and Tools Table, Materials and Methods section

Antibodies	Information included in the manuscript?	In which section is the information available? (Reagents and Tools Table, Materials and Methods, Figures, Data Availability Section)
For **antibodies** provide the following information:<br>- Commercial antibodies: RRID (if possible) or supplier name, catalogue number and or/clone number<br>- Non-commercial: RRID or citation	Yes	Reagents and Tools Table, Materials and Methods section

DNA and RNA sequences	Information included in the manuscript?	In which section is the information available? (Reagents and Tools Table, Materials and Methods, Figures, Data Availability Section)
**Short novel DNA or RNA including primers, probes**: provide the sequences.	Yes	Reagents and Tools Table, Materials and Methods section

Cell materials	Information included in the manuscript?	In which section is the information available? (Reagents and Tools Table, Materials and Methods, Figures, Data Availability Section)
**Cell lines:** Provide species information, strain. Provide accession number in repository **OR** supplier name, catalog number, clone number, and/**OR** RRID.	Yes	Reagents and Tools Table, Materials and Methods section
**Primary cultures:** Provide species, strain, sex of origin, genetic modification status.	Not Applicable	
Report if the cell lines were recently **authenticated** (e.g., by STR profiling) and tested for mycoplasma contamination.	Not Applicable	

Experimental animals	Information included in the manuscript?	In which section is the information available? (Reagents and Tools Table, Materials and Methods, Figures, Data Availability Section)
**Laboratory animals or Model organisms:** Provide species, strain, sex, age, genetic modification status. Provide accession number in repository **OR** supplier name, catalog number, clone number, **OR** RRID.	Yes	Reagents and Tools Table, Materials and Methods section
**Animal observed in or captured from the field:** Provide species, sex, and age where possible.	Not Applicable	
Please detail **housing and husbandry conditions**.	Yes	Materials and Methods section

Plants and microbes	Information included in the manuscript?	In which section is the information available? (Reagents and Tools Table, Materials and Methods, Figures, Data Availability Section)
**Plants:** provide species and strain, ecotype and cultivar where relevant, unique accession number if available, and source (including location for collected wild specimens).	Not Applicable	
**Microbes:** provide species and strain, unique accession number if available, and source.	Not Applicable	

Human research participants	Information included in the manuscript?	In which section is the information available? (Reagents and Tools Table, Materials and Methods, Figures, Data Availability Section)
If collected and within the bounds of privacy constraints report on age, sex and gender or ethnicity for all study participants.	Not Applicable	

Core facilities	Information included in the manuscript?	In which section is the information available? (Reagents and Tools Table, Materials and Methods, Figures, Data Availability Section)
If your work benefited from core facilities, was their service mentioned in the acknowledgments section?	Yes	Acknowledgement section

Design

Study protocol	Information included in the manuscript?	In which section is the information available? (Reagents and Tools Table, Materials and Methods, Figures, Data Availability Section)
If study protocol has been **pre-registered, provide DOI in the manuscript**. For clinical trials, provide the trial registration number **OR** cite DOI.	Not Applicable	
Report the **clinical trial registration number** (at ClinicalTrials.gov or equivalent), where applicable.	Not Applicable	

Laboratory protocol	Information included in the manuscript?	In which section is the information available? (Reagents and Tools Table, Materials and Methods, Figures, Data Availability Section)
Provide DOI OR other citation details if **external detailed step-by-step protocols** are available.	Not Applicable	

Experimental study design and statistics	Information included in the manuscript?	In which section is the information available? (Reagents and Tools Table, Materials and Methods, Figures, Data Availability Section)
Include a statement about **sample size** estimate even if no statistical methods were used.	Yes	Materials and Methods section
Were any steps taken to minimize the effects of subjective bias when allocating animals/samples to treatment (e.g. **randomization procedure**)? If yes, have they been described?	Yes	Materials and Methods section
Include a statement about **blinding** even if no blinding was done.	Yes	Materials and Methods section
Describe **inclusion/exclusion criteria** if samples or animals were excluded from the analysis. Were the criteria pre-established?<br><br>If sample or data points were omitted from analysis, report if this was due to attrition or intentional exclusion and provide justification.	Not Applicable	
For every figure, are **statistical tests** justified as appropriate? Do the data meet the assumptions of the tests (e.g., normal distribution)? Describe any methods used to assess it. Is there an estimate of variation within each group of data? Is the variance similar between the groups that are being statistically compared?	Yes	Materials and Methods section

Sample definition and in-laboratory replication	Information included in the manuscript?	In which section is the information available? (Reagents and Tools Table, Materials and Methods, Figures, Data Availability Section)
In the figure legends: state number of times the experiment was **replicated** in laboratory.	Yes	Figure legends
In the figure legends: define whether data describe **technical or biological replicates**.	Yes	Figure legends

Ethics

Ethics	Information included in the manuscript?	In which section is the information available? (Reagents and Tools Table, Materials and Methods, Figures, Data Availability Section)
Studies involving **human participants**: State details of **authority granting ethics approval** (IRB or equivalent committee(s), provide reference number for approval.	Not Applicable	
Studies involving **human participants**: Include a statement confirming that **informed consent** was obtained from all subjects and that the experiments conformed to the principles set out in the WMA Declaration of Helsinki and the Department of Health and Human Services Belmont Report.	Not Applicable	
Studies involving **human participants:** For publication of **patient photos**, include a statement confirming that consent to publish was obtained.	Not Applicable	
Studies involving experimental **animals**: State details of **authority granting ethics approval** (IRB or equivalent committee(s), provide reference number for approval. Include a statement of compliance with ethical regulations.	Yes	Materials and Methods section
Studies involving **specimen and field samples:** State if relevant **permits** obtained, provide details of authority approving study; if none were required, explain why.	Not Applicable	

Dual Use Research of Concern (DURC)	Information included in the manuscript?	In which section is the information available? (Reagents and Tools Table, Materials and Methods, Figures, Data Availability Section)
Could your study fall under dual use research restrictions? Please check biosecurity documents and list of **select agents and toxins** (CDC): https://www.selectagents.gov/sat/list.htm	Not Applicable	
If you used a select agent, is the security level of the lab appropriate and reported in the manuscript?	Not Applicable	
If a study is subject to dual use research of concern regulations, is the name of the **authority granting approval and reference number** for the regulatory approval provided in the manuscript?	Not Applicable	

Reporting

The MDAR framework recommends adoption of discipline-specific guidelines, established and endorsed through community initiatives. Journals have their own policy about requiring specific guidelines and recommendations to complement MDAR.

Adherence to community standards	Information included in the manuscript?	In which section is the information available? (Reagents and Tools Table, Materials and Methods, Figures, Data Availability Section)
State if relevant guidelines or checklists (e.g., **ICMJE, MIBBI, ARRIVE, PRISMA**) have been followed or provided.	Not Applicable	
For **tumor marker prognostic studies,** we recommend that you follow the **REMARK** reporting guidelines (see link list at top right). See author guidelines, under 'Reporting Guidelines'. Please confirm you have followed these guidelines.	Not Applicable	
For **phase II and III randomized controlled trials**, please refer to the **CONSORT** flow diagram (see link list at top right) and submit the CONSORT checklist (see link list at top right) with your submission. See author guidelines, under 'Reporting Guidelines'. Please confirm you have submitted this list.	Not Applicable	

Data Availability

Data availability	Information included in the manuscript?	In which section is the information available? (Reagents and Tools Table, Materials and Methods, Figures, Data Availability Section)
Have **primary datasets** been deposited according to the journal's guidelines (see 'Data Deposition' section) and the respective accession numbers provided in the Data Availability Section?	Yes	Data Availability section
Were **human clinical and genomic datasets** deposited in a public access-controlled repository in accordance to ethical obligations to the patients and to the applicable consent agreement?	Not Applicable	
Are **computational models** that are central and integral to a study available without restrictions in a machine-readable form? Were the relevant accession numbers or links provided?	Not Applicable	
If publicly available data were reused, provide the respective **data citations in the reference list**.	Not Applicable	
