# Supplementary material for: FBXL21 regulates diurnal proteostasis in skeletal muscle by targeting DNAJB6 and client proteins
Source: EMBO Rep. 2026 Sep 30;27(21):6366–95. doi: 10.1038/s44319-026-00922-1 (PMC13743554; doi:10.1038/s44319-026-00922-1)
Supplement: Supplementary file 2 [file 44319_2026_922_MOESM8_ESM.pdf]

Expanded View Figures

Figure EV1. Identification of DNAJB6 as a novel target of FBXL21.

(**A**) Yeast two-hybrid (Y2H) assay demonstrating the interaction between FBXL21 and DNAJB6. As a positive control, yeast cells were co-transformed with pGBKT7-p53 (bait) and pGADT7-SV40 (prey), expressing the Gal4 DNA-binding domain (BD) fused to murine p53 and the Gal4 activation domain (AD) fused to the SV40 large T antigen, respectively. (**B**, **C**) Co-immunoprecipitation assays showing the interaction between FBXL21 and human DNAJB6a or DNAJB6b. (**D**) mRNA expression of *Dnajb6* in WT and *Psttm* mice. Data were presented as mean ± SEM ($n = 5$/group/time point). No mark indicates not statistically significant. (**E**) Immunoblot of DNAJB6 in 0.5% Triton X-100 soluble and insoluble fractions from WT and *Psttm* mice. GAPDH was used as a positive control for the soluble fraction. Data were presented as mean ± SEM ($n = 3$ mice/group/time point). ***$P = 0.0002$ and ****$P < 0.0001$; Two-way ANOVA with Tukey's multiple comparisons test, comparing between WT and *Psttm* mice or between time points. ###$P = 0.0002$ and ####$P < 0.0001$; Unpaired Student's *t*-test indicates significant differences between specific comparisons. (**F**) Immunoblot analysis of FBXL21 expression in Cntl and *Fbxl21* KO C2C12 cells. Data were presented as mean ± SEM ($n = 3$). *$P = 0.0363$, Student's *t*-test, indicating a significant difference between groups. (**G**) Immunofluorescence staining of endogenous FBXL21 in Cntl and *Fbxl21* KO C2C12 cells by confocal microscopy. Scale bar: 20 µm. (**H**) Negative control image (secondary antibody only) for Fig. 1F. *N* numbers indicate biological replicates for each sample group, and representative data from three independent experiments are shown.

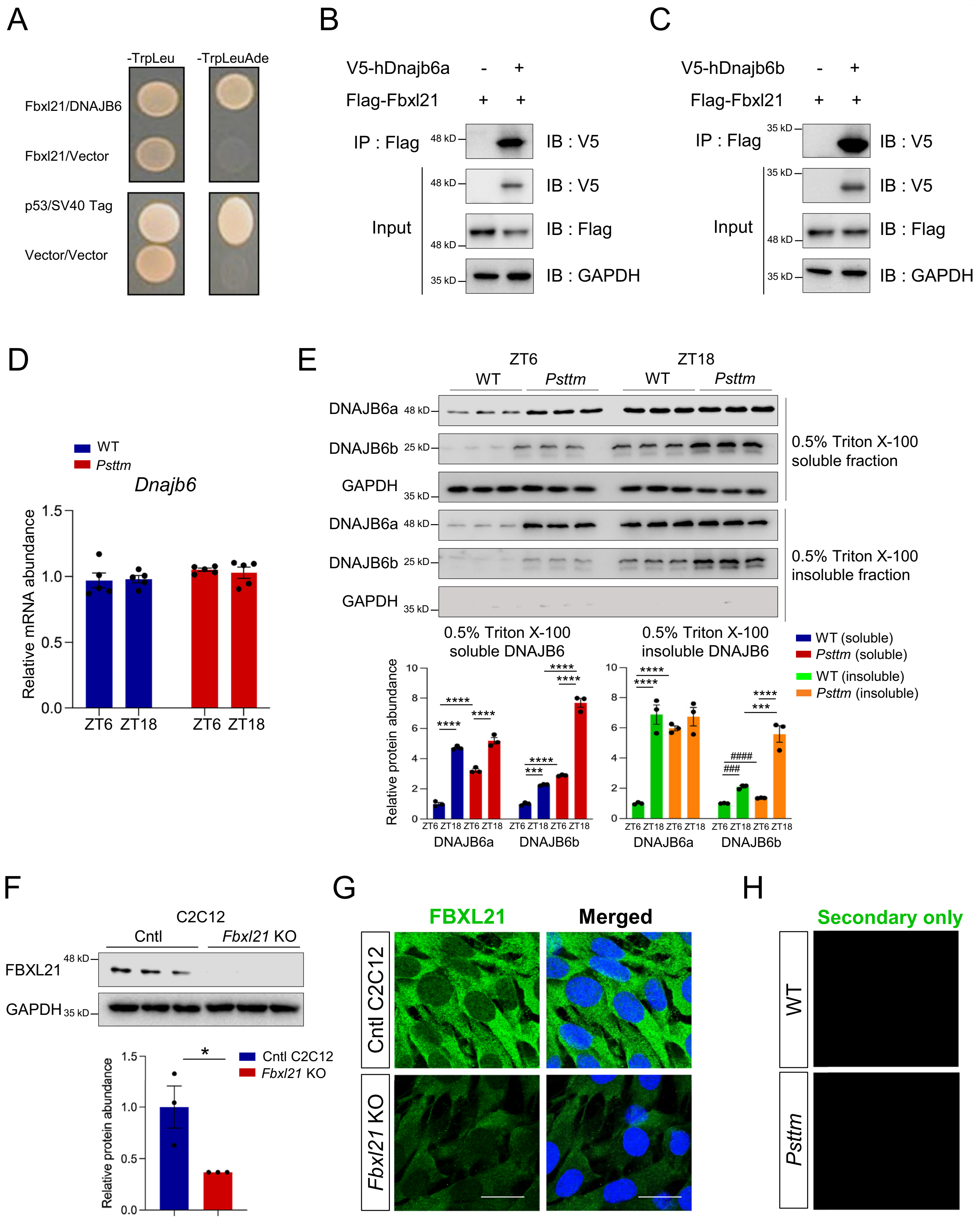
A
-TrpLeu
-TrpLeuAde
Fbxl21/DNAJB6
Fbxl21/Vector
p53/SV40 Tag
Vector/Vector
B
V5-hDnajb6a - +
Flag-Fbxl21 + +
IP : Flag
48 kD
IB : V5
Input
48 kD
IB : V5
IB : Flag
48 kD
35 kD
IB : GAPDH
C
V5-hDnajb6b - +
Flag-Fbxl21 + +
IP : Flag
35 kD
IB : V5
Input
35 kD
IB : V5
IB : Flag
48 kD
35 kD
IB : GAPDH
D
WT
Psttm
Dnajb6
Relative mRNA abundance
1.5
1.0
0.5
0.0
ZT6 ZT18 ZT6 ZT18
E
ZT6
ZT18
WT
Psttm
DNAJB6a 48 kD
DNAJB6b 25 kD
GAPDH 35 kD
0.5% Triton X-100 soluble fraction
0.5% Triton X-100 insoluble fraction
0.5% Triton X-100 soluble DNAJB6
0.5% Triton X-100 insoluble DNAJB6
Relative protein abundance
WT (soluble)
Psttm (soluble)
WT (insoluble)
Psttm (insoluble)
****
***
####
###
ZT6 ZT18 ZT6 ZT18
DNAJB6a
DNAJB6b
F
C2C12
Cntl
Fbxl21 KO
FBXL21 48 kD
GAPDH 35 kD
Relative protein abundance
Cntl C2C12
Fbxl21 KO
*
1.5
1.0
0.5
0.0
G
FBXL21
Merged
Cntl C2C12
Fbxl21 KO
H
Secondary only
WT
Psttm

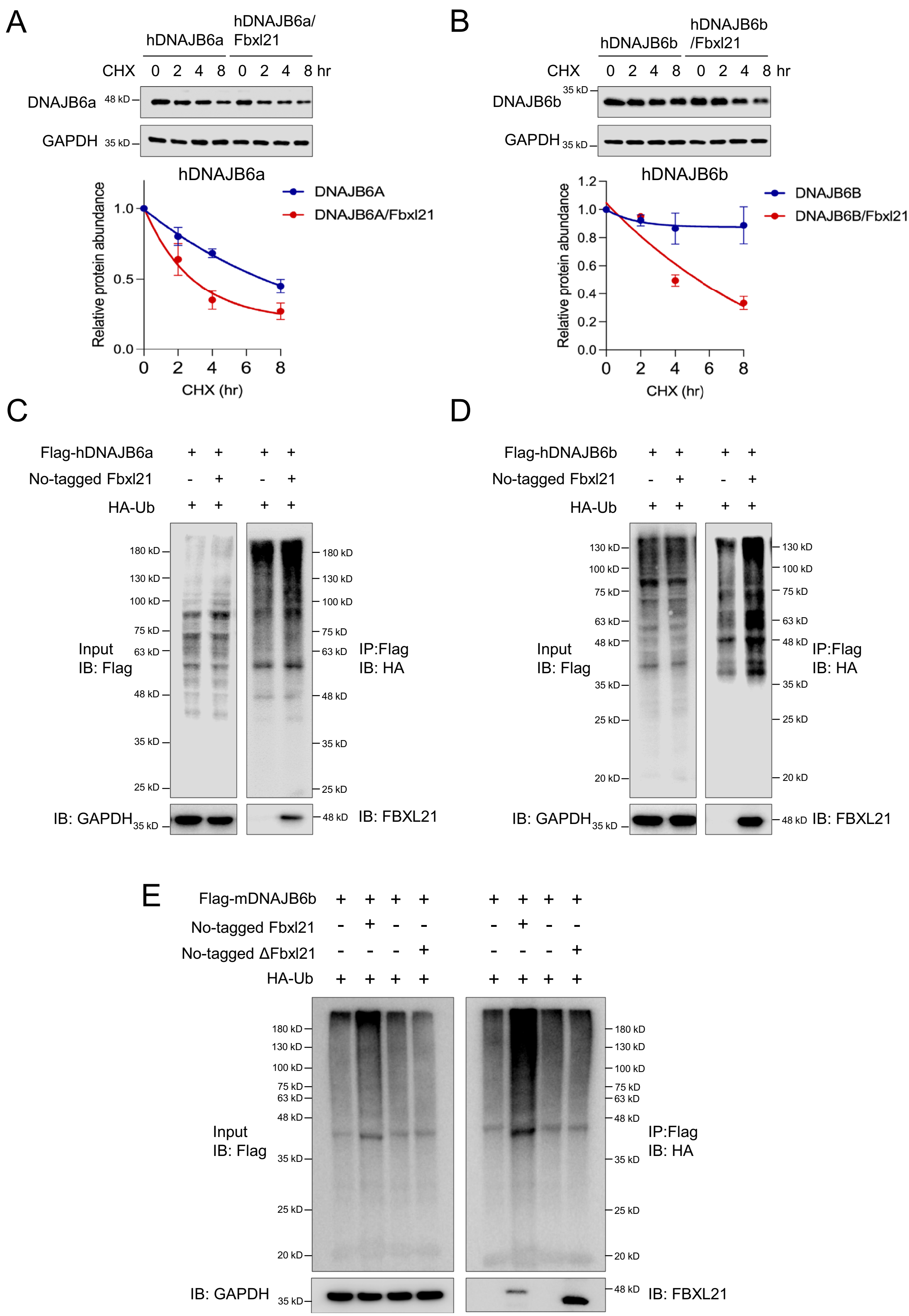
A
hDNAJB6a
hDNAJB6a/ Fbxl21
CHX 0 2 4 8 0 2 4 8 hr
DNAJB6a 48 kD
GAPDH 35 kD
hDNAJB6a
DNAJB6A
DNAJB6A/Fbxl21
Relative protein abundance
CHX (hr)
B
hDNAJB6b
hDNAJB6b /Fbxl21
CHX 0 2 4 8 0 2 4 8 hr
DNAJB6b 35 kD
GAPDH 35 kD
hDNAJB6b
DNAJB6B
DNAJB6B/Fbxl21
Relative protein abundance
CHX (hr)
C
Flag-hDNAJB6a + + + +
No-tagged Fbxl21 - + - +
HA-Ub + + + +
Input
IB: Flag
IP:Flag
IB: HA
IB: GAPDH
IB: FBXL21
D
Flag-hDNAJB6b + + + +
No-tagged Fbxl21 - + - +
HA-Ub + + + +
Input
IB: Flag
IP:Flag
IB: HA
IB: GAPDH
IB: FBXL21
E
Flag-mDNAJB6b + + + + + + + +
No-tagged Fbxl21 - + - - - + - -
No-tagged ΔFbxl21 - - - + - - - +
HA-Ub + + + + + + + +
Input
IB: Flag
IP:Flag
IB: HA
IB: GAPDH
IB: FBXL21

Figure EV2. FBXL21 regulates degradation and ubiquitination of human DNAJB6 isoforms.

(**A**, **B**) Cycloheximide (CHX) chase assays were performed to assess the protein stability of human DNAJB6a (**A**) and DNAJB6b (**B**) in 293T cells. Protein half-lives were quantified. Half-lives: hDnajb6a, 7.05 h; hDnajb6a/Fbxl21, 2.82 h; hDnajb6b, >24 h; hDnajb6b/Fbxl21. Data were presented as mean ± SEM ($n = 3$). (**C**, **D**) FBXL21 promotes the ubiquitination of human DNAJB6 isoforms. Flag-tagged DNAJB6a (**C**) or DNAJB6b (**D**) was co-transfected with untagged FBXL21 and HA-tagged ubiquitin (HA-Ub), followed by ubiquitination assays. (**E**) ΔFBXL21 abolished its ability to ubiquitinate DNAJB6b in 293T cells. Flag-tagged mDNAJB6b was co-transfected with either wild-type FBXL21 or ΔFBXL21 with HA-tagged ubiquitin (HA-Ub), followed by ubiquitination assays. *N* numbers indicate biological replicates for each sample group, and representative data from three independent experiments are shown.

 © The Author(s)

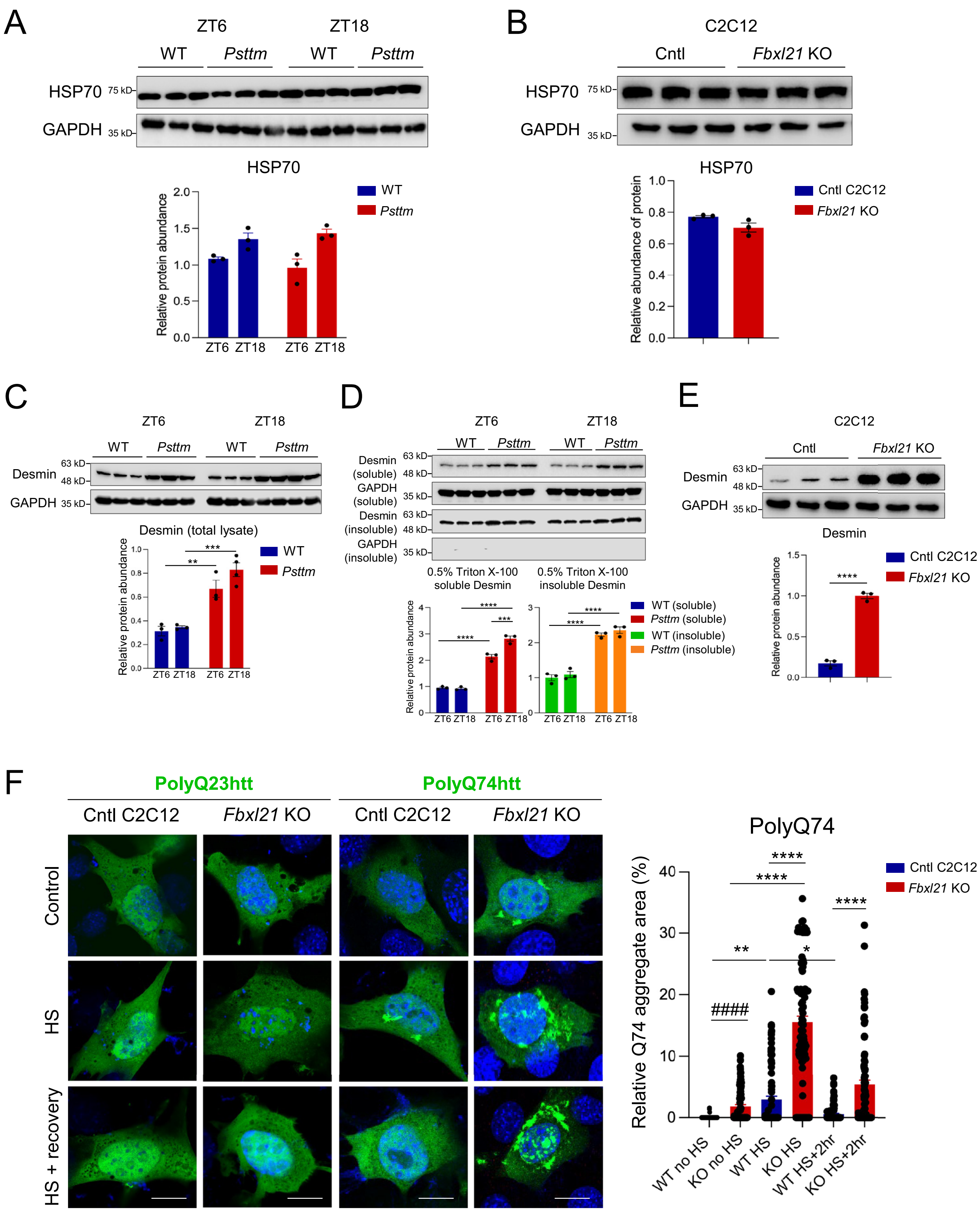
A
ZT6
ZT18
WT
Psttm
WT
Psttm
HSP70 75 kD
GAPDH 35 kD
HSP70
WT
Psttm
Relative protein abundance
ZT6 ZT18 ZT6 ZT18
B
C2C12
Cntl
Fbxl21 KO
HSP70 75 kD
GAPDH 35 kD
HSP70
Cntl C2C12
Fbxl21 KO
Relative abundance of protein
C
Desmin 63 kD 48 kD
GAPDH 35 kD
Desmin (total lysate)
D
Desmin (soluble)
GAPDH (soluble)
Desmin (insoluble)
GAPDH (insoluble)
0.5% Triton X-100 soluble Desmin
0.5% Triton X-100 insoluble Desmin
WT (soluble)
Psttm (soluble)
WT (insoluble)
Psttm (insoluble)
E
Desmin
F
PolyQ23htt
PolyQ74htt
Cntl C2C12
Fbxl21 KO
Control
HS
HS + recovery
PolyQ74
Relative Q74 aggregate area (%)
WT no HS
KO no HS
WT HS
KO HS
WT HS+2hr
KO HS+2hr

Figure EV3. FBXL21 regulates desmin proteostasis and polyQ74htt aggregation without altering HSP70 expression.

(**A**) Immunoblot analysis of HSP70 protein levels in wild-type (WT) and *Psttm* mice collected at Zeitgeber time 6 (ZT6; daytime) and ZT18 (nighttime). GAPDH was used as a loading control. Data were presented as mean ± SEM ($n = 3$/group/time point). No mark indicates not statistically significant. $P = 0.7027$ (WT ZT6 vs. *Psttm* ZT6) and $P = 0.8803$ (WT ZT18 vs. *Psttm* ZT18); Two-way ANOVA with Tukey's multiple comparisons test. (**B**) Immunoblot analysis of HSP70 protein levels in control and *Fbxl21* KO C2C1 cells. Data were presented as mean ± SEM ($n = 3$). No mark indicates not statistically significant. $P = 0.0816$; Unpaired Student's *t*-test. (**C**) Immunoblot analysis of Desmin protein levels in WT and *Psttm* mice. GAPDH was used as a loading control. Data were presented as mean ± SEM ($n = 3$–4). **$P = 0.0059$ and ***$P = 0.0004$; two-way ANOVA indicates statistical differences between genotypes (WT vs. *Psttm*). (**D**) Immunoblot of Desmin in 0.5% Triton X-100 soluble and insoluble fractions from WT and *Psttm* mice. GAPDH was used as a positive control for the soluble fraction. Data were presented as mean ± SEM ($n = 3$). ***$P = 0.0001$ and ****$P < 0.0001$; Two-way ANOVA with Tukey's multiple comparisons test, comparing between WT and *Psttm* mice or between time points. (**E**) Immunoblot analysis of Desmin protein levels in Cntl and *Fbxl21* KO C2C12 cells. GAPDH was used as a loading control. Data were presented as mean ± SEM ($n = 3$). ****$P < 0.0001$; Unpaired Student's *t*-test. (**F**) Confocal microscopy analysis in Cntl and *Fbxl21* KO C2C12 cells expressing polyQ23htt-EGFP and polyQ74htt-EGFP. PolyQ23htt-EGFP was used as a negative control, and no aggregates were detected in the groups. The relative Q74 aggregate area (%) was quantified using ImageJ. Data were presented as mean ± SEM ($n = 100$ cells/group). *$P = 0.0253$ (WT HS vs. WT HS recovery), **$P = 0.0023$ (WT no HS vs. WT HS), and ****$P < 0.0001$; One-way ANOVA showing significant differences between groups. ####$P < 0.0001$; Unpaired Student's *t*-test. *N* numbers indicate biological replicates for each sample group, and representative data from three independent experiments are shown. Scale bar: 15 µm.

 © The Author(s)

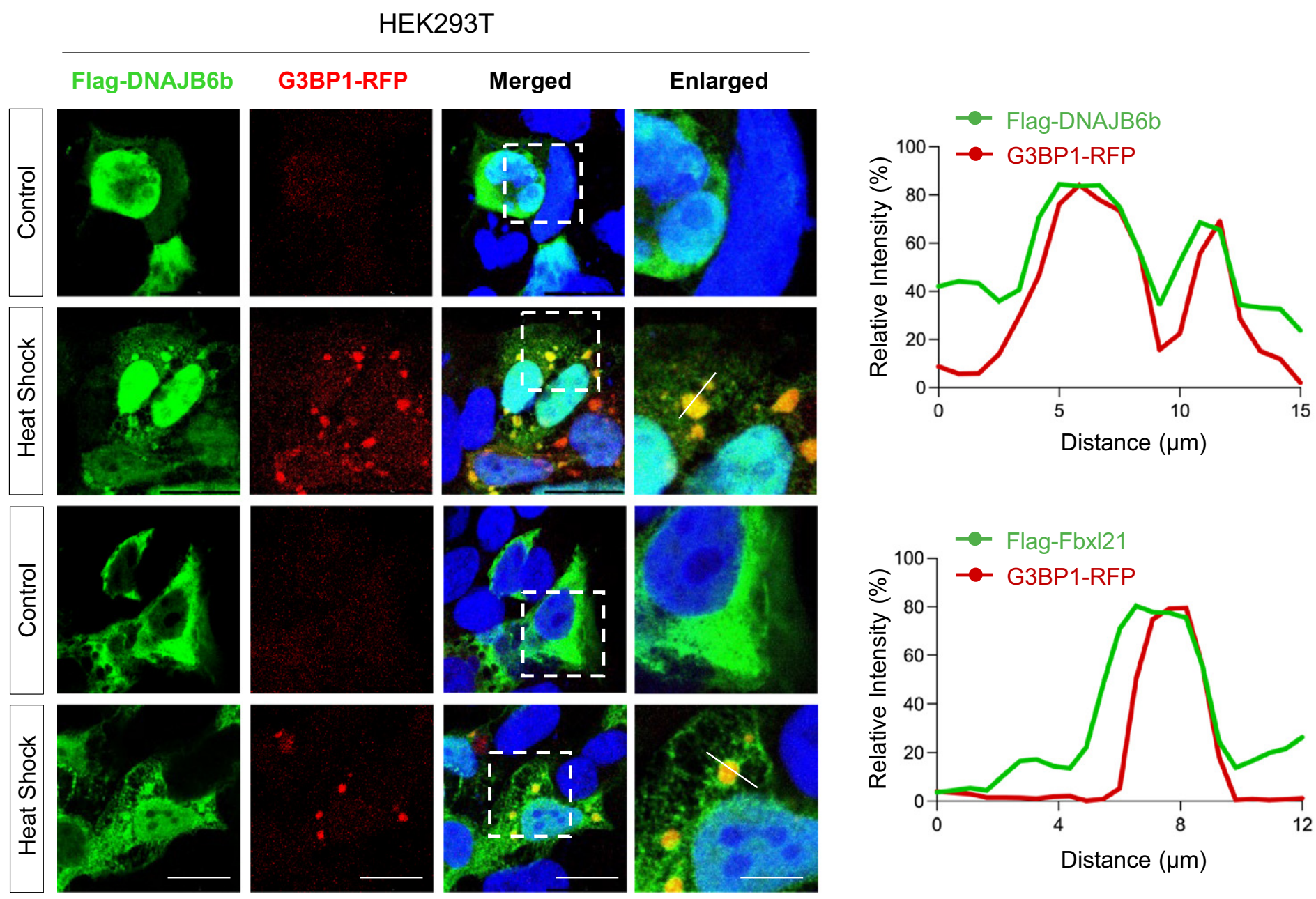

Figure EV4. DNAJB6 and FBXL21 colocalize in stress granules.

Left panel: Confocal microscopy of HEK293T cells co-expressing either Flag-DNAJB6b and G3BP1-RFP or Flag-FBXL21 and G3BP1-RFP. Scale bar: 20 μm. Right panels: Line profiles showing the mean fluorescence intensities analyzed by using Prism. Representative images are shown from three independent experiments are shown. Scale bars: 10 μm (enlarged images) and 20 μm (all other images).

© The Author(s)

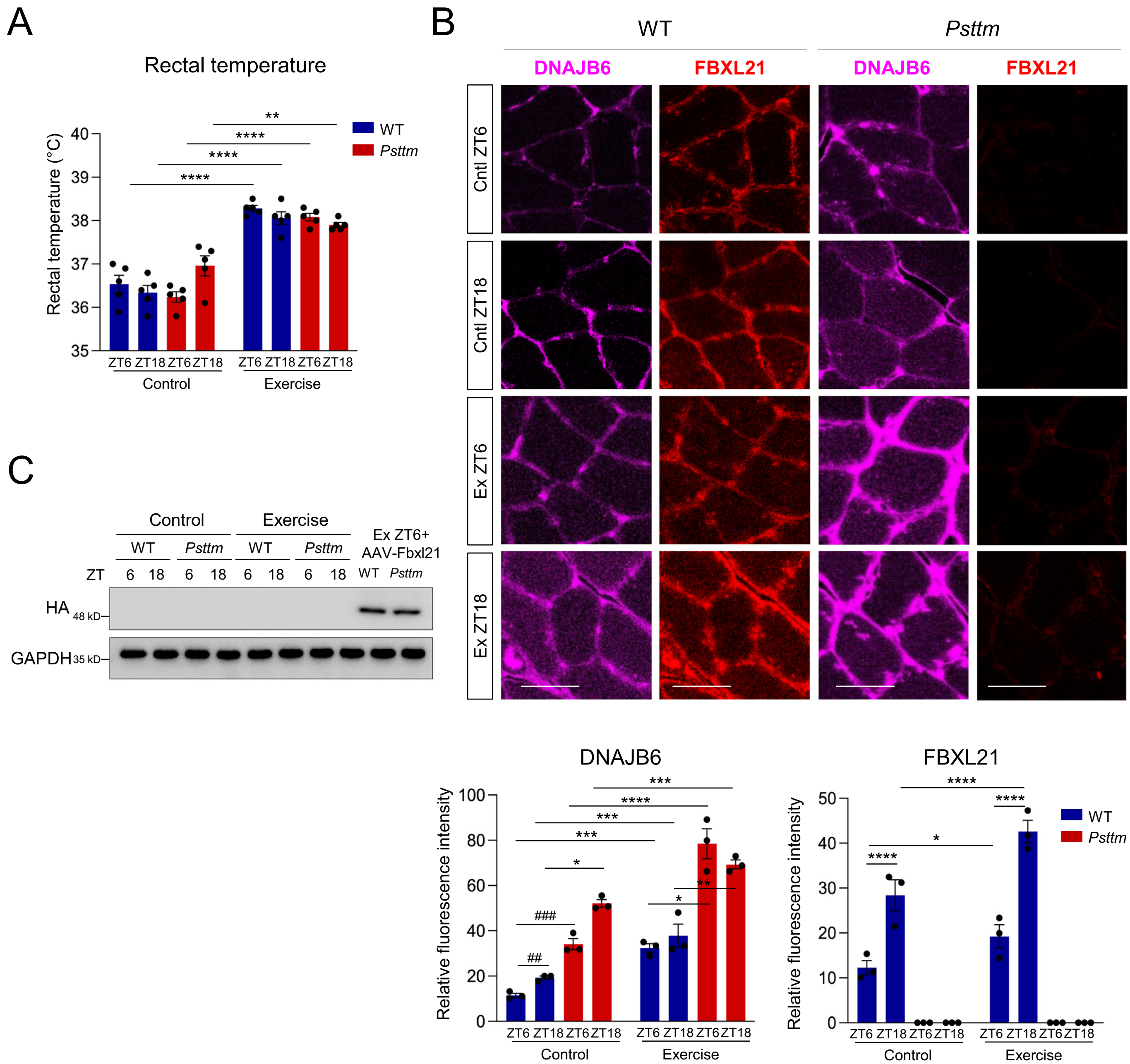

Figure EV5. Exercise alters DNAJB6 and FBXL21 expression levels in WT and *Psttm* mice in a time-dependent manner.

(**A**) Rectal temperature measured using a rectal probe (ThermoWorks) at different time points. Data were presented as mean ± SEM ($n = 5$ mice/group/time point). No mark indicates not statistically significant. $^{**}P = 0.0017$ and $^{****}P < 0.0001$, Two-way ANOVA with Tukey's multiple comparisons test, comparing between groups. (**B**) Immunofluorescence staining of DNAJB6 and FBXL21 in gastrocnemius muscle cross-sections from wild-type (WT) and *Psttm* mice under control (non-exercise) conditions. Scale bar: 20 µm. Lower left panel: Quantification of DNAJB6 fluorescence intensity in gastrocnemius muscle cross-sections from WT and *Psttm* mice under control and acute exercise conditions. Lower right panel: Quantification of FBXL21 fluorescence intensity in gastrocnemius muscle cross-sections from WT and *Psttm* mice under control and acute exercise conditions. Data were presented as mean ± SEM ($n = 3$/group/time point). For DNAJB6, $^{*}P = 0.0483$ (control WT ZT18 vs. control *Psttm* ZT18), $^{*}P = 0.0131$ (exercise WT ZT6 vs. exercise *Psttm* ZT6), $^{**}P = 0.0061$ (exercise WT ZT18 vs. exercise *Psttm* ZT18), $^{***}P = 0.0015$ (control WT ZT6 vs. exercise WT ZT6), $^{***}P = 0.0001$ (exercise WT ZT18 vs. exercise *Psttm* ZT18), and $^{****}P < 0.0001$; Two-way ANOVA with Tukey's multiple comparisons test. $^{\#\#}P = 0.0028$ and $^{\#\#\#}P = 0.0010$; Unpaired Student's *t*-test. For FBXL21, $^{*}P = 0.0176$ and $^{****}P < 0.0001$; Two-way ANOVA with Tukey's multiple comparisons test. (**C**) Immunoblot assay for HA detection in gastrocnemius tissues of WT and *Psttm* mice subjected to daytime exercise and AAV-CK6-Fbxl21-HA injection.

 © The Author(s)
